# Assessing Population Diversity of *Brettanomyces* Yeast Species and Identification of Strains for Brewing Applications

**DOI:** 10.3389/fmicb.2020.00637

**Published:** 2020-04-09

**Authors:** Marc Serra Colomer, Anna Chailyan, Ross T. Fennessy, Kim Friis Olsson, Lea Johnsen, Natalia Solodovnikova, Jochen Forster

**Affiliations:** ^1^Carlsberg Research Laboratory, Group Research, Copenhagen, Denmark; ^2^National Institute for Food, Technical University of Denmark, Kongens Lyngby, Denmark; ^3^MS-Omics ApS, Vedbaek, Denmark

**Keywords:** genomics, high-throughput screening, brewing fermentation, phenolic off-flavor, 4-ethylguaiacol, maltose assimilation, beta-glucosidase, *Dekkera bruxellensis*

## Abstract

*Brettanomyces* yeasts have gained popularity in many sectors of the biotechnological industry, specifically in the field of beer production, but also in wine and ethanol production. Their unique properties enable *Brettanomyces* to outcompete conventional brewer’s yeast in industrially relevant traits such as production of ethanol and pleasant flavors. Recent advances in next-generation sequencing (NGS) and high-throughput screening techniques have facilitated large population studies allowing the selection of appropriate yeast strains with improved traits. In order to get a better understanding of *Brettanomyces* species and its potential for beer production, we sequenced the whole genome of 84 strains, which we make available to the scientific community and carried out several *in vitro* assays for brewing-relevant properties. The collection includes isolates from different substrates and geographical origin. Additionally, we have included two of the oldest Carlsberg Research Laboratory isolates. In this study, we reveal the phylogenetic pattern of *Brettanomyces* species by comparing the predicted proteomes of each strain. Furthermore, we show that the *Brettanomyces* collection is well described using similarity in genomic organization, and that there is a direct correlation between genomic background and phenotypic characteristics. Particularly, genomic patterns affecting flavor production, maltose assimilation, beta-glucosidase activity, and phenolic off-flavor (POF) production are reported. This knowledge yields new insights into *Brettanomyces* population survival strategies, artificial selection pressure, and loss of carbon assimilation traits. On a species-specific level, we have identified for the first time a POF negative *Brettanomyces anomalus* strain, without the main spoilage character of *Brettanomyces* species. This strain (CRL-90) has lost *DaPAD1*, making it incapable of converting ferulic acid to 4-ethylguaiacol (4-EG) and 4-ethylphenol (4-EP). This loss of function makes CRL-90 a good candidate for the production of characteristic *Brettanomyce*s flavors in beverages, without the contaminant increase in POF. Overall, this study displays the potential of exploring *Brettanomyces* yeast species biodiversity to find strains with relevant properties applicable to the brewing industry.

## Introduction

In 1904, the Danish scientist Niels Hjelte Claussen isolated and identified a new yeast species at the Carlsberg Research Laboratory (CRL—formerly Carlsberg Laboratory). The yeast was named “*Brettanomyces*” meaning “British fungus,” after being found in an English stock ale beer. Curiously, *Brettanomyces* was then the first microorganism in the history being patented (Claussen, 1904, 1906). In continuity to Claussen’s work, Holger Schiønning characterized more *Brettanomyces* isolates who he named “Torula” between the years 1905 and 1908 (Schiønning, 1908; Andersion, 2012). From then, *Brettanomyces* species have been isolated in wineries and breweries all over the world, as well as other substrates like sodas, olives, kombucha, and bioethanol production plants (Schifferdecker et al., 2014; Smith and Divol, 2016). In its sexual form, it is also referred to as *Dekkera*, a genus comprising the most frequently found species *Dekkera/Brettanomyces bruxellensis* and *Dekkera/Brettanomyces anomalus* (for review, see Smith, 2011). In addition, other asexual species of *Brettanomyces* have been described, such as *Brettanomyces naardenensis*, *Brettanomyces custersianus*, and *Brettanomyces nanus* (Kurtzman et al., 2011; Tiukova et al., 2019; Roach and Borneman, 2020). The potential of *Brettanomyces* species for brewing is controversial, as it is usually recognized as a spoilage yeast, being the cause of major economic losses in many production facilities (Gilliland, 1961; Lebleux et al., 2020). Its main spoilage feature is the production of phenolic off-flavor (POF), more specifically 4-ethylguaiacol (4-EG) and 4-ethylphenol (4-EP), imparting displeasing characters to the final product, described as horsy, barnyard, leather, band-aid, or medicine (Heresztyn, 1986; Chatonnet et al., 1992; Callemien and Collin, 2010). Nevertheless, *Brettanomyces* species are playing an important role in spontaneously fermented traditional Belgian beers, such as Lambics or Gueuzes, and are also contributing with “funky” characters to farmhouse ales (Spitaels et al., 2014; Crauwels et al., 2015a; Steensels et al., 2015). Over the last decade, the craft beer sector has constantly demanded novel attractive flavors, and there has been a rising interest in understanding *Brettanomyces* species, exploiting its potential in beer fermentation and ethanol production (for review, see Colomer et al., 2019).

Understanding the relationship between genotype and phenotype is essential for strain selection and optimization of brewing. However, the lack of genomic tools to perform gene deletions in *Brettanomyces* strains is still a bottleneck to establish convincing genotype-phenotype correlations (Miklenić et al., 2015; Varela et al., 2018). Moreover, while yeast breeding via sporulation and mating is a common practice for strain improvement in brewer’s yeast (Sanchez et al., 2012; Steensels et al., 2014; Gibson et al., 2017), it is still unclear if there is such a tool available for *Brettanomyces* species. To date, several assemblies of *B. bruxellensis* species have been published (Curtin et al., 2012; Piškur et al., 2012; Borneman et al., 2014; Crauwels et al., 2014; Olsen et al., 2015; Fournier et al., 2017) displaying a wide intra-specific variability of *B. bruxellensis* species (Woolfit et al., 2007; Hellborg and Piškur, 2009; Borneman et al., 2014). There are frequent variations at genomic level, including re-organizations and gene duplications, with the number of chromosomes varying from 4 to 9 (Woolfit et al., 2007; Gounot et al., 2019). While most of the population is diploid (2n) or triploid (3n) (Borneman et al., 2014; Avramova et al., 2018a), up to five alleles for certain locus have been reported (Avramova et al., 2018a). In addition, there is an increasing number of studies focusing on the human influence on the domestication of conventional brewer’s yeast suggesting that yeast populations have been pushed toward a concrete phenotype: POF negative, high flocculation, maltose assimilation (Gallone et al., 2016; Gonçalves et al., 2016; Preiss et al., 2017; Peter et al., 2018). Artificial selection pressure has also been investigated for *Brettanomyces* species, mainly with the usage of SO_2_ as a wine preservative. Recent studies have found a correlation between a triploid state and SO_2_ tolerance (Curtin and Pretorius, 2014; Avramova et al., 2018b). Interestingly, another recent study, using microsatellite genotyping of 1488 different *B. bruxellensis* isolates concluded that ploidy explains much of the population variance and can be correlated to SO_2_ tolerance (Avramova et al., 2018a).

The biochemical pathways involved in beer fermentation and aroma formation in brewer’s yeasts have been extensively studied. Fewer studies have been done in *Brettanomyces* yeasts, although genetic similarities with brewer’s yeasts have been reported (Curtin and Pretorius, 2014). One of them is the use of promoter rewiring for survival strategies, based on producing, accumulating, and consuming ethanol (Ihmels et al., 2005; Rozpedowska et al., 2011). A crucial trait for an efficient beer fermentation is maltose utilization. The MAL locus is commonly duplicated as an adaptive response to the substrate (Needleman et al., 1984; Vanoni et al., 1989; Gallone et al., 2016). While complex regulatory pathways involved in maltose utilization have been characterized in brewer’s yeast (Jespersen et al., 1999; Horák, 2013), maltose metabolism in *Brettanomyces* is still unclear. Different sugar consumption patterns have been reported, with variations in maltose and maltotriose utilization (Crauwels et al., 2017; Smith and Divol, 2018). Ethanol production is closely linked to sugar utilization, and *Brettanomyces* has been widely described as a potential bioethanol producer (Blomqvist et al., 2010; Aguilar-Uscanga et al., 2011; Schifferdecker et al., 2016). A unique property of *Brettanomyces* yeasts is their high β-glucosidase activity, conferring the capability to break beta linked substrates (Daenen et al., 2004, 2008b). This activity allows the release of aromatic monoterpene alcohols from hops, enhancing “flowery” and “citrus” characters in beer. Additionally, it facilitates *Brettanomyces* long-lasting survival in wooden barrels by enhancing the break-down of cellobiose, the main disaccharide present in wood (for review, see Steensels et al., 2015). Two ORFs coding for β-glucosidases have been identified, and other non-specific exo-glucanases (EXG) have been suggested (Crauwels et al., 2014; Kuo et al., 2018).

Quantification of volatile compounds (VOCs) is a common practice to predict the flavor and aroma of a beverage. VOCs are secondary metabolites produced by the yeast during fermentation, generally consisting of higher alcohols, esters, ketones, and phenols. Higher alcohols are commonly related to solvent-like aromas and are mainly products of the catabolism of aromatic amino acids (leucine, isoleucine, valine) through the Ehrlich pathway (Hazelwood et al., 2008). Esters are associated with pleasant fruity flavors and comprise two types: acetate esters and ethyl esters. Acetate esters are normally synthesized with acetyl-CoA reacting with ethanol or a higher alcohol, the most frequent being isoamyl acetate (banana aroma), ethyl acetate (lipstick aroma), and 2-phenylacetate (honey aroma) (Bisson and Karpel, 2010). These reactions are catalyzed by either Atf1 or Atf2, and can be reversed by the action of Iah1 esterase (Fujii et al., 1994; Fukuda et al., 2000). As a result, the ratio between higher alcohols and acetate esters remains dependent on the balance of Atf1/Iah1 activities (Fukuda et al., 1998). Ethyl esters are described as pineapple, grape, and tropical fruit aromas and are formed in a reaction of an activated fatty acid with ethanol by the activity of ethanol acyl transferases: Eeb1 and Eht1 (Saerens et al., 2006). In addition, formation of the “buttery” off-flavor diacetyl during alcoholic fermentation presents a challenge for brewers. Diacetyl is formed by a non-enzymatic reaction from acetolactate leaking out of the cell. Synthesis of acetolactate is mediated by the *ILV2* and *ILV6* genes among others (Gjermansen et al., 1988; Duong et al., 2011). As acetolactate is an intermediate in the branched-chain amino acids (BCAA) biosynthetic pathways, it can be feedback regulated by valine (Krogerus and Gibson, 2013). Furthermore, POF production by brewer’s yeasts has been well characterized. 4-Vinylguaiacol (4-VG) production is a result of a dimeric interaction of Pad1 and Fdc1 proteins decarboxylating ferulic acid from the media (Mukai et al., 2010). In *Brettanomyces* species, POF production consists of a two-step conversion pathway, with an additional reduction step of 4-VG to 4-EG (Heresztyn, 1986; Godoy et al., 2009). A phenolic acid decarboxylase (BbPad1) has been identified as being responsible for the decarboxylation of ferulic acid (Godoy et al., 2014), and a superoxide dismutase (BbSod) has been suggested for the second reduction step (4-VG to 4-EG) (Romano et al., 2017). The same pathway is used to produce 4-vinylphenol (4-VP) and 4-EP, using coumaric acid as a substrate. The proportion of 4-EG/4-EP in *Brettanomyces* fermented *beer* is 3:1, while in wine it is 1:1 or lower (for review, see Crauwels et al., 2015a). As 4-EP is generally more unpleasant than 4-EG, such ratio could play a significant role in explaining why *Brettanomyces* species are more undesired in wine (Crauwels et al., 2015a; Lentz and Harris, 2015; Cibrario et al., 2020).

In order to gain a better understating of the genetic diversity and the brewing capabilities of *Brettanomyces*, we sequenced the whole genome of 84 strains including the species *B. bruxellensis, B. anomalus*, *B. custersianus*, and *B*. *naardenensis*, derived from different sources worldwide, and make them available to the scientific community. The genomes were annotated, the function of each gene was predicted, and the strains were compared with each other. Strains were grouped according to the genomic organization, absence or presence of brewing-relevant genes was investigated. Essential brewing traits were investigated, including wort fermentation and flavor analysis along with *in vitro* assays to predict specific enzymatic activity. Finally, the genotype–phenotype relationship has been investigated, revealing new population trends and highlighting new application areas of *Brettanomyces*.

## Materials and Methods

### *Brettanomyces* Strain Collection and Culture Conditions

**FIGURE 1:**
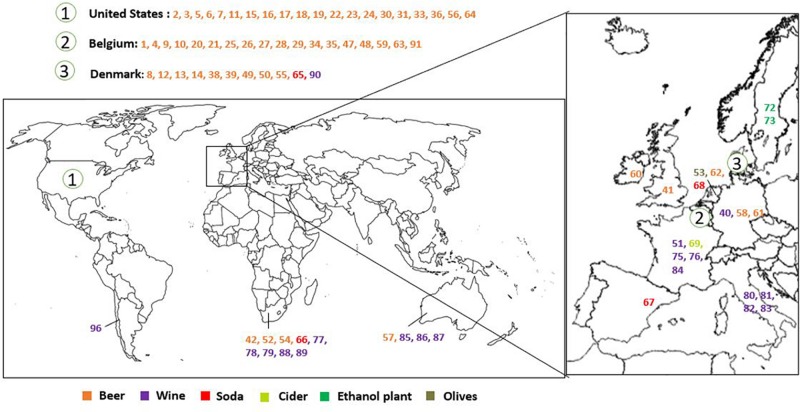
Geographical distribution and substrate of origin of the *Brettanomyces* strains included in the study.

*Brettanomyces* strains were selected from CRL yeast strain collection. Strains used in this study are mainly isolated from beer. Isolates from wine, kombucha, sodas, olives, and bioethanol production plants, among others, are also included. The geographical origin of the strains is very diverse, comprising isolates from Europe, South America, Africa, and Australia (Figure 1 and Table 1). We have included two of the oldest known *Brettanomyces* isolates in the world, obtained at the CRL in Denmark between the years 1904–1908 (CRL-49, CRL-50) (Schiønning, 1908).

**TABLE 1:** List of *Brettanomyces* strains included in the study.

**CRL num**	**Specie**	**Source**	**Country**	**Collection ID**
CRL-1	*B. bruxellensis*	Beer	Belgium	*Brettanomyces* Drie BSI
CRL-2	*B. bruxellensis*	Cask beer	United States	–
CRL-3	*B. bruxellensis*	Cask beer	United States	–
CRL-4	*B. bruxellensis*	Beer	Belgium	Wyeast5112
CRL-5	*B. bruxellensis*	Beer	United States	WLP648
CRL-6	*B. bruxellensis*	Beer	United States	WLP650
CRL-7	*B. anomalus*	Beer	United States	Wyeast 5151
CRL-8	*B. bruxellensis*	Beer	Denmark	WLP645
CRL-9	*B. bruxellensis*	Beer	Belgium	Wyeast5526
CRL-10	*B. bruxellensis*	Beer	Belgium	WLP653
CRL-11	*B. bruxellensis*	Cask beer	United States	-
CRL-12	*B. bruxellensis*	Cask beer	Denmark	-
CRL-13	*B. bruxellensis*	Beer	Denmark	-
CRL-14	*B. bruxellensis*	Beer	Denmark	-
CRL-15	*B. bruxellensis*	Saison beer	United States	-
CRL-16	*B. bruxellensis*	Beer	United States	American Farmhouse-Imperial yeast
CRL-17	*B. bruxellensis*	Beer	United States	Sour Batch Kidz-Imperial Yeast isolate
CRL-18	*B. anomalus*	Beer	United States	Sour Batch Kidz-Imperial Yeast isolate
CRL-19	*B. bruxellensis*	Beer	United States	Suburban Brett-Imperial Yeast
CRL-20	*B. bruxellensis*	Beer	Belgium	Saccharolicious BrettI
CRL-21	*B. bruxellensis*	Beer	Belgium	Saccharolicious BrettII
CRL-22	*B. bruxellensis*	Beer	United States	–
CRL-23	*B. bruxellensis*	Beer	United States	Belgian sour mix—The Yeast Bay
CRL-24	*B. bruxellensis*	Beer	United States	Belgian sour mix—The Yeast Bay
CRL-25	*B. bruxellensis*	Beer	Belgium	–
CRL-26	*B. bruxellensis*	Beer	Belgium	Brussels blend—The Yeast Bay
CRL-27	*B. bruxellensis*	Abbey Beer	Belgium	Orval beer
CRL-28	*B. bruxellensis*	Beer	Belgium	–
CRL-29	*B. bruxellensis*	Beer	Belgium	–
CRL-30	*B. anomalus*	Beer	United States	Amalgamation—The Yeast Bay
CRL-31	*B. bruxellensis*	Beer	United States	Amalgamation—The Yeast Bay
CRL-33	*B. bruxellensis*	Beer	United States	Amalgamation—The Yeast Bay
CRL-34	*B. bruxellensis*	Beer	Belgium	Beersel Blend—The Yeast Bay
CRL-35	*B. bruxellensis*	Beer	Belgium	Lochristi Blend—The Yeast Bay
CRL-36	*B. bruxellensis*	India pale ale	United States	–
CRL-38	*B. bruxellensis*	Cask beer	Denmark	–
CRL-39	*B. anomalus*	Lambic beer	Denmark	–
CRL-40	*B. bruxellensis*	Wine	Germany	CBS2796
CRL-41	*B. anomalus*	Beer	United Kingdom	CBS77
CRL-42	*B. custersianus*	Beer	South Africa	CBS5207
CRL-47	*B. bruxellensis*	Beer	Belgium	CBS74
CRL-48	*B. bruxellensis*	Beer	Belgium	CBS75
CRL-49	*B. anomalus*	Beer—CRL (1906)	Denmark	CBS76
CRL-50	*B. bruxellensis*	Beer—CRL (1906)	Denmark	CBS78
CRL-51	*B. bruxellensis*	Wine	France	CBS2336
CRL-52	*B. bruxellensis*	Bantu beer	South Africa	CBS5512
CRL-53	*B. custersianus*	Olives	Netherlands	CBS8347
CRL-54	*B. custersianus*	Bantu beer	South Africa	CBS4805
CRL-55	*B. bruxellensis*	Beer	Denmark	–
CRL-56	*B. bruxellensis*	India pale ale	United States	–
CRL-57	*B. anomalus*	Beer	Australia	CBS1947
CRL-58	*B. anomalus*	Beer	Germany	CBS4712
CRL-59	*B. bruxellensis*	Lambic beer	Belgium	CBS72
CRL-60	*B. bruxellensis*	Porter beer	Ireland	CBS96
CRL-61	*B. anomalus*	Beer	Germany	CBS4711
CRL-62	*B. bruxellensis*	Stout beer	Netherlands	CBS98
CRL-63	*B. bruxellensis*	Beer	Belgium	–
CRL-64	*B. naardenensis*	Soda water	United States	CBS6040
CRL-65	*B. naardenensis*	Lemonade	Denmark	CBS6108
CRL-66	*B. naardenensis*	Soft drink	South Africa	CBS7540
CRL-67	*B. anomalus*	Soft drink	Spain	–
CRL-68	*B. anomalus*	Soft drink	Netherlands	CBS8138
CRL-69	*B. anomalus*	Cider	France	CBS4212
CRL-72	*B. bruxellensis*	Ethanol plant	Sweden	CBS1292
CRL-73	*B. bruxellensis*	Ethanol plant	Sweden	CBS1315
CRL-74	*B. anomalus*	Sherry vat	South Africa	CBS4608
CRL-75	*B. bruxellensis*	Sour wine	France	CBS1943
CRL-76	*B. bruxellensis*	Sour wine	France	CBS2547
CRL-77	*B. bruxellensis*	Wine	South Africa	CBS4602
CRL-78	*B. bruxellensis*	Champagne	South Africa	CBS4481
CRL-79	*B. bruxellensis*	Sherry wine	South Africa	CBS4482
CRL-80	*B. bruxellensis*	Wine	Italy	–
CRL-81	*B. bruxellensis*	Wine	Italy	–
CRL-82	*B. bruxellensis*	Wine	Italy	–
CRL-83	*B. bruxellensis*	Wine	Italy	–
CRL-84	*B. bruxellensis*	Wine	France	–
CRL-85	*B. bruxellensis*	Wine	Australia	–
CRL-86	*B. bruxellensis*	Wine	Australia	–
CRL-87	*B. bruxellensis*	Wine	Australia	–
CRL-88	*B. bruxellensis*	Wine	South Africa	–
CRL-89	*B. bruxellensis*	Wine	South Africa	–
CRL-90	*B. anomalus*	Wine	Denmark	–
CRL-91	*B. bruxellensis*	Beer	Belgium	–
CRL-96	*B. bruxellensis*	Wine	Chile	–

*All strains are pure cultures isolated from different substrates and geographical locations.*

For strain isolation from beverages, Wallerstein Laboratory Nutrient (WLN) agar containing bromocresol green supplemented with cycloheximide (4 μg/mL) was used as a selection media. *Brettanomyces* strains were differentiated based on color and morphology when metabolizing bromocresol green. Preliminary species differentiation was performed by polymerase chain reaction (PCR) amplification of the internal transcriber spacer (ITS) region followed by gel electrophoresis (Fujita et al., 2001). In the current study, 84 isolates were used, comprising 64 *D. bruxellensis*, 14 *D. anomalus*, three *B. naardenensis*, and three *B. custersianus*. For general cultivation, strains were grown in yeast peptone dextrose (YPD) at 25°C with agitation (100 r/min, IKA KS 501 digital). For storage purposes, YPD supplemented with 20% glycerol was used to freeze the cultures at −80°C. To perform high-throughput phenotyping, the strain collection was arranged in triplicates in Greiner Flat-Bottom Microtiter Plate (MTP, 96-well format). Biomek FXP Automated Workstation (Beckman–Coulter) was employed for general liquid handling and for *in vitro* assays.

### Yeast Whole-Genome Sequencing

For genomic DNA isolation, single colonies were inoculated in liquid YPD and grown for 1 week in 50 mL shake flasks. Biomass was collected by centrifugation (4000 *g*, 10 min, 4°C), washed with sterile water, and stored at −4°C in 70% ethanol. Pellets were delivered for DNA extraction, short insert size library preparation (<800 bp), 150 bp paired-end sequencing on Illumina HiSeq4000 whole-genome sequencing (performed by BGI Europe^®^). In order to get high-quality genome assemblies, representatives for each species, were selected for PACBio RSII technology long-read sequencing (CRL-49 as *B. anomalus*, CRL-53 as *B. custersianus*, and CRL-65 as *B. naardenensis*) (performed by BGI Europe^®^). For the *B. bruxellensis* group, the publicly available genome of the isolate UMY321 was obtained from NCBI (Fournier et al., 2017).

### Bioinformatics Analysis: Genome Assembly, Gene Calling, Prediction of Function, and Phylogenetic Comparison

*De novo* assembly for all the 84 yeast strains was performed using ABySS (Assembly By Short Sequences), a parallelized sequence assembler. Strain species were identified by blasting against the internal transcribed spacer (ITS) region from fungi type and reference material database of NCBI.

The PacBio raw sequencing reads were assembled with Canu software (Koren et al., 2017) using the default parameters. Each Canu assembly was used as a reference to map the corresponding short reads using fast and versatile mapper (Minimap2) (Li, 2018). The resulting SAM files were transformed into sorted and indexed BAM files using Samtools (Li et al., 2009). Finally, we used a tool for polishing our Canu assemblies with the Illumina short reads to produce an improved draft assembly for each of the PacBio sequenced yeast.

We next divided the whole pool into four groups (according to its species) and for each one of them defined the reference. For the *B. anomalus* species, the “polished” sequence of CRL-49 was used as a reference; the CRL-53 was used as a reference for the group *B. custersianus*, while the CRL-65 served as a reference for the *B. naardenensis* group. The genome of the *D. bruxellensis* UMY321 strain served as a reference for the *B/D. bruxellensis* group (Fournier et al., 2017). It is publicly available from NCBI and the authors obtained the most complete and contiguous *de novo* assembly of *D. bruxellensis* using a combination of Nanopore long-read and Illumina short-read sequencing data (Fournier et al., 2017). For all the four groups, we mapped the short Illumina reads to the reference using BWA-MEM algorithm and set a filter on MAPQ (mapping quality) 30, in order to get only uniquely mapped reads. We then generated a consensus sequence for each genome which was used for the downstream analyses.

For gene prediction, we used Augustus with the consensus sequence generated on the mapping step (Stanke et al., 2006). The *Saccharomyces cerevisiae* S288C was used as the closest related species. For each yeast, the amino acid sequence and the general feature file (GFF) were saved for the later steps. To predict the function of each open reading frame (ORF), a query against the Protein Database of NCBI with keywords “brettanomyces, saccharomyces, saccharomycopsis” was done, resulting in 1,318,613 hits, which were then used to build a BLAST DB (functional_db). Each of the proteins of the three references (CRL-49, CRL-53, CRL-65) were searched against the functional_db using an e-value cut-off of 1e-4 and in the case of success the function was then transferred from the hit to the query.

To compare the 85 yeast genomes, OrthoFinder, a platform for comparative genomics was used (Emms and Kelly, 2015, 2019). Orthogroups, gene orthologs, and complete set of gene tree were computed from raw amino acid sequences with OrthoFinder. The species tree was inferred from the set of unrooted orthogroup gene trees, and a consensus tree was taken of all individual estimates using STAG algorithm (Emms and Kelly, 2018), and was rooted using STRIDE (Emms and Kelly, 2017). The metadata consisting of “Source,” “Species,” and “Continent” was added in the dataset, and the phylogenetic tree was imported on CLC Genomics Workbench 11 software (QIAGEN Bioinformatics^®^). The tree visualization was elaborated under the Circular cladogram and radial display (QIAGEN, 2016). CLC Genomics Software was used for general genome analysis and data handling. BLAST function on CLC was used to predict regions of loss, and hits scoring > 95% of the total BLAST score were considered as valid for gene presence.

### Phenotypic Characterization and *in vitro* Assays

#### Small-Scale Wort Fermentation

Yeast strains were propagated in triplicates in YPD media in 96-well plate format (Greiner Bio-one^®^) at room temperature with agitation at 100 r/min for 5 days. Cultures were diluted 1:20 in water prior to inoculation. 10 μL of the yeast dilution was inoculated into standard pilsner wort (Viking malt^®^) for a final volume of 270 μL/well; 96 well-plates (CR1496c—Enzyscreen^®^) were sealed with anaerobic lids and kept at 25°C with agitation at 200 r/min in a GrowthProfiler 960 (Enzyscreen^®^), with pictures automatically taken every 30 min to monitor cell growth. To obtain a relative growth value, pictures were converted to G-Value using the image analysis software (Enzyscreen). Fermentations were harvested after 7 days, cells were removed by centrifugation (3000 *g*, 4°C, 5 min), and supernatant was kept for further analysis.

#### Determination of Ethanol and Acetic Acid in Beers

Quantification of ethanol and acetic acid was performed in 96-well format following indicated microplate assay procedure. In order to quantify ethanol content, samples were diluted 1:200 in water and determined using the Ethanol Assay Kit K-ETOH^®^ from Megazyme. In order to quantify acetic acid content, samples were diluted 1:100 in water and determined using Acetic Acid (Acetate Kinase Manual Format) K-ACETRM^®^ from Megazyme. Both kits are based on stoichiometric reactions using NADH as a cofactor and measurements in the decrease/increase of absorbance at 340 nm (Megazyme^®^). A calibration curve of known concentrations was performed for the accurate determination of each compound. Optical density was measured in a Spark^®^Multimode Plate Reader (TECAN).

#### Determination of Volatile Compounds in Beer With Gas Chromatography/Mass Spectrometry

The main flavor-active compounds produced by the *Brettanomyces* collection were analyzed using gas chromatography/mass spectrometry analysis. The beer sample size was 200 μL. Analytes were extracted using liquid–liquid extraction with methyl-tert-butyl ether directly in the vial. The analysis was performed using a mid-polarity column (Zebron^TM^ ZB-1701, GC Cap. Column 30 m × 0.25 mm × 0.25 μm) installed in a GC (7890B, Agilent) coupled with a quadrupole detector (59977B, Agilent). The system was controlled by ChemStation (Agilent). The GC-method was set up as described by Pinu and Villas-Boas (2017). Raw data were converted to netCDF format using Chemstation (Agilent), before the data were imported and processed in Matlab R2014b (Mathworks, Inc.). Quantifications were performed using external calibration lines.

#### Assay for Utilization of Glucose and Maltose With Biolog

Cells were grown in 96-well plates in YPD until saturation. Cultures were diluted in sterile water 1:10^4^ before inoculation in the appropriate media. Media consisted of Yeast Nitrogen Base with amino acids supplemented with 1% glucose and maltose, respectively, as a carbon source. Strains were inoculated in triplicates into Biolog^®^ 96-well plates and incubated at 25°C without agitation. Growth kinetics was monitored with OmniLog^®^Biolog. The quantification was coupled with formation of the purple formazan due to reduction of the tetrazolium dye by metabolically active cells. The quantification of the purple formazan formation was performed by Omnilog^®^Biolog software and its output is given in B values. Data handling was performed using OmniLog_FileManagement Software.

#### Assay for Growth on Cellobiose and Extracellular β-Glucosidase Activity

*Brettanomyces* strains were inoculated in triplicates into 96-well plates containing Yeast Peptone Cellobiose (2% Cellobiose) and incubated for 1 week at 25°C. Absorbance was measured at 600 nm to monitor growth. Cultures were spun down and supernatant was collected to measure extracellular β-glucosidase activity. The enzymatic beta-glucosidase test was performed with MAK129^®^ β-glucosidase assay kit from Sigma–Aldrich. P-nitrophenyl-β-D-glucopyranoside (β-NPG) was used as a substrate and variation of optical density was measured at 405 nm after 20 min incubation at 37°C. Absorbance on 96-well plate format was measured on Spark^®^Multimode Plate Reader (TECAN). The assay results are given in units/L, one unit is the amount of enzyme that catalyzes the hydrolysis of 1.0 μmole substrate per minute at pH = 7.

#### Screening for POF and Quantification of Volatile Phenols With UPLC

Phenolic off-flavor production was screened using an absorbance-based method based on the uptake of ferulic acid (Steensels, 2017). Yeast cells were grown in triplicates in 96-well plates with YPD media for 7 days. Cultures were diluted 1:100 in water before inoculation in a new 96-well plate with YPD supplemented with ferulic acid at 0.1 mg/mL. After 1 week cultivation at 25°C with agitation (100 r/min, IKA KS 501 digital), plates were centrifuged (4000 *g*, 5 min, 4°C), 100 μL of supernatant was collected, and absorbance was measured at 325 nm using a Spark^®^Multimode Plate Reader (TECAN).

During wort fermentations, phenolic compounds (ferulic acid, coumaric acid, 4-EG, 4-EP) were quantified by ultra performance liquid chromatography (UPLC) (Waters) with PDA detection (280 nm). The separation was achieved using the BEH Phenyl Ultra Column (2.1 × 100 mm, 1.7 μm) and a flow of 0.5 mL/min. The injection volume was 1 μL. The mobile phase was 99.9% A, 0.1% B between 0 and 3 min. followed by a gradient up to 45% B for 5 min. Eluent A was contained 3% formic acid, 10% methanol in water and eluent B was 100% methanol. Calibration standards were prepared in methanol in the range 0.1–10 mg/L by dilution of a 10 mg/L standard mix. Beer samples were filtered on a 0.2 μm filter, diluted 1.5× with eluent A and vortexed for 5 s. Compounds were identified by retention time and ID was confirmed by spiking with standard solution.

### Intermediate Fermentations

Strains were propagated in pilsner wort in 50 mL Erlenmeyer shake flasks. A pitching rate of 100,000 cells/mL was determined using a Cellometer X2 (Nexelom Bioscience) to count the cells. Fermentations were performed in duplicates in 250 mL Duran bottles containing 200 mL of standard pilsner wort (Viking Malt). Cumulative pressure was monitored with ANKOM RF Gas Production System^®^ (ANKOM). Fermentations were stopped after 7 days, cells were removed by centrifugation (4000 *g*, 10 min, 4°C) and the supernatant was used for analysis.

### Principal Component Analysis and Statistical Analysis

Principal component analysis (PCA) was performed on all 84 individuals, described by 12 variables. PCA was performed using the R software (version 3.5.2). Missing values were addressed by imputing them using the imputePCA function. PCA was performed using first five principal components, which explained 75% of the variance. A biplot displaying PC scores of different strains (points) and loadings for each variable (cos2-vector) was produced. Variables are colored by cos2 (squared coordinates) high cos2 indicates a good representation, a low-bad. The colors are: red = good, green = bad. Observations are shown as balls, which are colored according the grouping following the Genetic.Cluster. For each group, there is a bigger ball representing the mean of the group (centroid). Correlation and PCA scores were extracted and included. Statistical analysis was performed on phenotypical data and *in vitro* analysis, with one-way ANOVA followed by Tukey *post hoc* test (*p* < 0.05) using R software (version 3.5.2). The detailed output of the statistical analysis can be found at Supplementary Material (Statistical tests).

## Results

### Genetic Clustering of *Brettanomyces* Species

**FIGURE 2:**
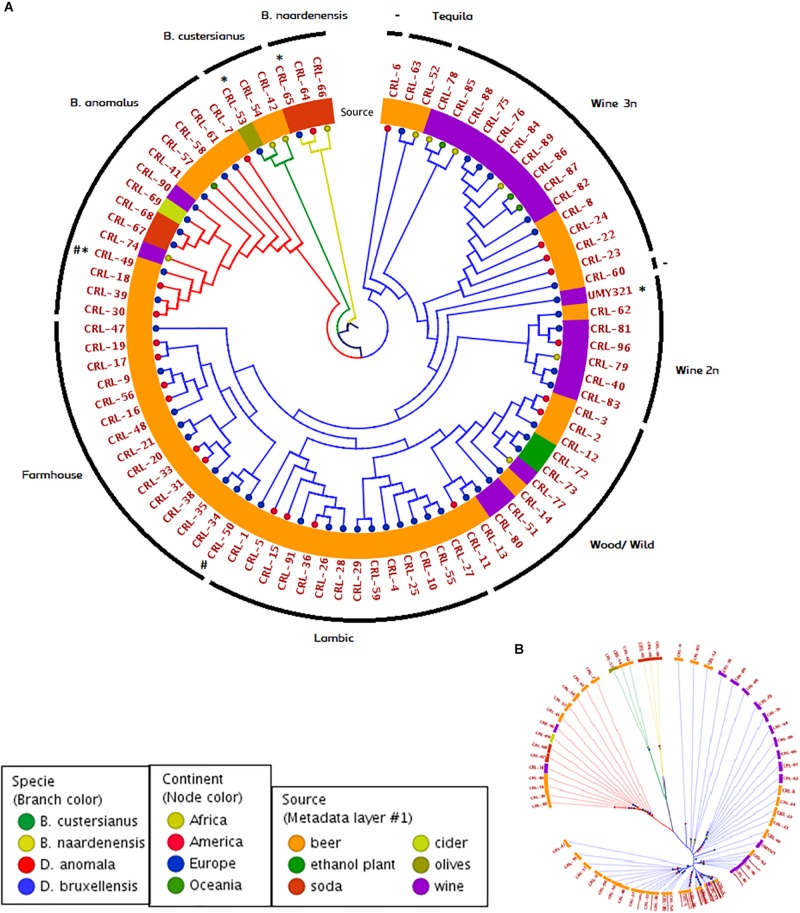
Genomic phylogeny of *Brettanomyces* population. **(A)** Circular claudogram of 84 *Brettanomyces* species included in this study. Phylogenetic tree was produced by comparison of the predicted proteins in the whole genome of each strain. Strains used as reference genome are marked with (^∗^) Old CRL isolates are marked with (#). Branches are colored according to species, tree nodes according to continent of origin, and metadata layer is colored according to substrate of isolation. Yeasts have been grouped in different genetic clusters according to its location in the tree and genetic cluster names are indicated in the external circle. **(B)** Same phylogenetic tree in radial form. Branch length and separation displays genomic divergence between *Brettanomyces* isolates.

In order to study the population structure of *Brettanomyces* genus, we sequenced the whole genome of 84 *Brettanomyces* strains, comprising the species *B. bruxellensis* (64), *B. anomalus* (14), *B. custersianus* (3), and *B. naardenensis* (3). Through comparison of protein sequences, a phylogenetic tree comparing all 84 isolates was constructed (Figure 2A). The different *Brettanomyces* species cluster together as shown by the branch color (Figure 2A). The metadata layer 1 displaying the substrate of isolation infers that the genomic set-up of *Brettanomyces* population is strongly influenced by the media they survive in. Interestingly, a major clade comprising strains isolated from beer is formed. This clade contains mainly commercial strains applied for craft brewing referred to as “Farmhouse” and another clade where the majority of strains are isolated from spontaneously fermented Belgian Lambic beers, which is labeled as “Lambic.” Moreover, a sub-clade within the “Lambic” group could be identified, where the strains come from various natural origins; ethanol plants, barrel-aged beers, and matured wines. Therefore, this sub-clade is labeled as “Wood/Wild.” The old Carlsberg *B. bruxellensis* strain CRL-50 is located at the edge of the “Farmhouse” cluster, indicating that after deposition this strain could have been applied for brewing purposes. With regard to the wine spoilage isolates, the genetic divergence is much higher as displayed by branch lengths in Figure 2B. Genetic similarities among beer strains are the first hint of artificial selection pressure, in which strains with desired phenotypes have been recently applied. It is observed that the geographical distribution of the beer isolates seems to be more homogenous than wine isolates. In contrast, the artificial selection of wine-spoilage *Brettanomyces* has occurred in a reversed way. The results indicate that strains have evolved in parallel in several wine regions in the world, developing resistance to wine preservatives, and resulting in higher genetic divergences (Curtin and Pretorius, 2014; Avramova et al., 2018a). In regard to wine isolates, two main clades are found. As previously described (Borneman et al., 2014; Avramova et al., 2018b), population structure of *B. bruxellensis* displays a variable ploidy of 2n/3n. The cluster “Wine 2n” comprises mainly diploid strains [also described as (CBS2499-like) in Avramova et al., 2018a], and the reference genome UMY321, while the cluster “Wine 3n” comprises mainly triploid strains (AWRI1499-like) in Avramova et al. (2018a). Moreover, there are some isolates highly divergent from the rest of strains, CRL-63, -52 labeled as (“Tequila”-like) in Avramova et al. (2018a) and showing potential signs to be the common ancestors in *B. bruxellensis* species.

### Chromosomal Organization of the Reference *B. bruxellensis* UMY321 Strain: Distribution of Functional Genes

**FIGURE 3:**
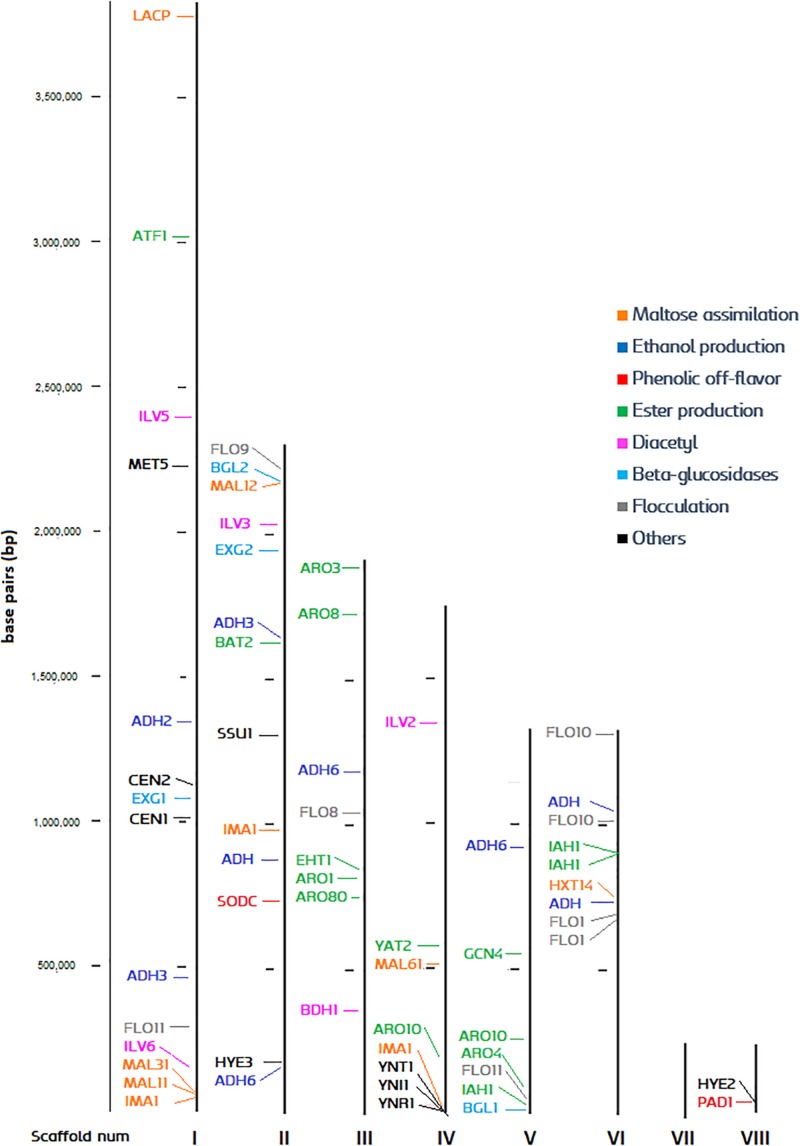
Distribution of brewing-relevant genes across the *B. bruxellensis* UMY321 reference genome.

The high-quality reference genome UMY321 was downloaded from NCBI (Fournier et al., 2017). The genome is organized in eight scaffolds, which could be considered as a chromosome level structure. All the ORFs of the genome were identified, and the function of each gene was predicted. The most relevant genes for brewing were pinpointed, and we produced a chromosome-like map with an overview of the genome distribution (Figure 3). The list of predicted gene function and its relevance is explained in Supplementary Table S1. Despite the genome of *Brettanomyces* being highly dynamic and karyotypes strongly variable (Hellborg and Piškur, 2009; Curtin and Pretorius, 2014), we could display an overview of the polymorphic subtelomeric regions where more variation is expected (Brown et al., 2010). The centromeric loci *CEN1*, *CEN2*, responsible for chromosome rearrangements and change of ploidy (Ishchuk et al., 2016) is located in scaffold I. Scaffold I also contains a maltose assimilation cluster (*MAL31*, *MAL11*, *IMA1*), similar to the one already described in brewer’s yeast in which is essential for maltose degradation (Needleman et al., 1984; Horák, 2013; Needleman and Michels, 2015). In this case, no transcriptional activator such as *ScMAL13* is present, but just an α-glucosidase surrounded by maltose permeases and transporters. The nitrate assimilation cluster is spotted in the subtelomeric region of scaffold IV, and both β-glucosidases (*BGL1*, *2*) are found on the edges of scaffolds V and II, respectively. *BbPAD1*, gene responsible for POF phenotype, is located at the shortest scaffold (VIII). As a general overview, most diacetyl, ester, and ethanol production genes are found at conserved regions, therefore low variability in these phenotypes could be expected. Furthermore, several events of gene duplication are present, especially abundant in scaffold VI. This phenomenon is commonly seen as a mechanism of adaptation by yeast (Hittinger and Carroll, 2007; Ames et al., 2010; Dunn et al., 2012; Gorter de Vries et al., 2017; Harari et al., 2018). For example, three repetitions of the major isomaltase IMA1 are present, displaying copy number repetitions to enhance utilization of carbon sources (Duval et al., 2010; Naumoff and Naumov, 2010; Teste et al., 2010). These numerous repetitions of *IMA1* are also common in brewing yeasts, with sequences sharing high similarities one to another (Libkind et al., 2011; Deng et al., 2014). *ADH6* responsible for aldehyde reduction (Larroy et al., 2002) is also present in triplicate along the genome and could have a potential role in environmental adaptation and *Brettanomyces* survival. Duplication of *ADH6* as a response to cell damage has been described in brewing yeast, as protein abundance increase when DNA-replication-stress is induced (Tkach et al., 2012). Moreover, three copies of isoamyl acetate esterase *IAH1* are noticed, which are presumably involved in acetate ester degradation (Fukuda et al., 2000; Colomer et al., 2019). The FLO gene family is often duplicated at the subtelomeric regions, a phenomenon already well-described in brewing yeast (Teunissen and Steensma, 1995). This gene family often show intrinsic tandem repeats and occasionally are transcriptionally silent (Van Mulders et al., 2009).

### Genomic Variability in *Brettanomyces* Populations

**FIGURE 4:**
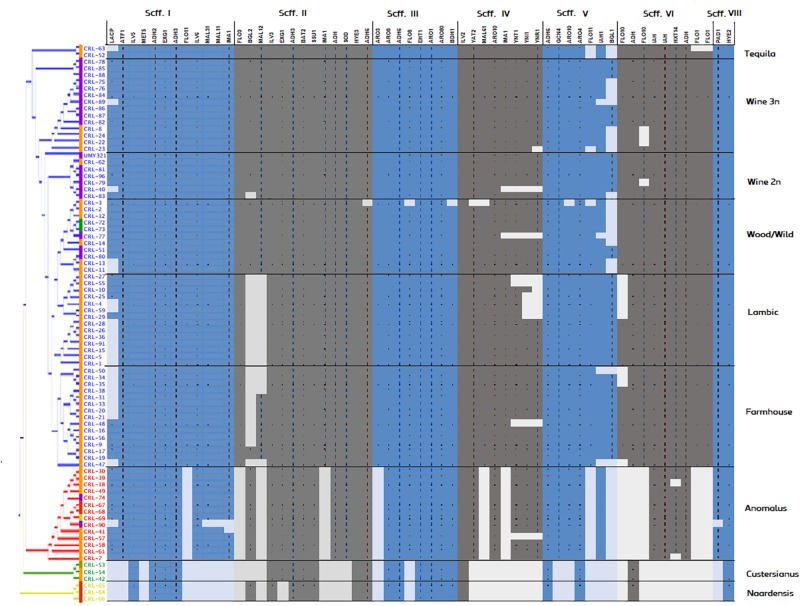
Genomic coverage of *Brettanomyces* strains included in the study. The reference genes spotted in Figure 3 were subtracted and a BLAST search was performed for each gene, respectively. To predict regions of loss, hits scoring > 95% of the total BLAST score were considered as valid for gene presence. Coloring in the genomic heatmap indicates absence/presence of the respective gene. Two strains showed a low read-mapping coverage and were removed from the figure (CRL-6, CRL-60).

A phylogenetic tree based on predicted functional ORFs was created with the aim of assessing genomic clustering among isolates (Figure 2). The identified genes from Figure 3 were used to perform a multi-BLAST search for each one of them, and a heatmap was created to assess major divergence among the previously classified genomic clusters (Figure 4). As the reference *B. bruxellensis* strain UMY321 is a diploid wine isolate strain, most of the “Wine 2n” cluster display a full genome coverage. Several strains of the “Wine 3n” cluster are missing an outer region of scaffold V, where *BGL1* is located among others. This is also the case in the “Wild/Wood” group. With regard to the beer isolates “Lambic” and “Farmhouse,” most isolates are missing the subtelomeric segments of scaffold I (where a sugar-transporter *LACP* is located) and scaffold II (*BGL2*-region). The lack of this β-glucosidase in beer-isolated strains has been observed before (Crauwels et al., 2015b). All strains in the “Lambic” sub-group are missing large internal region of the scaffold II and correspondingly lacking a maltase (*MAL12*). A high number of strains in the “Lambic” cluster are also missing the nitrogen assimilation cluster in the subtelomeric region of scaffold IV.

The “Anomalus,” “Custersianus,” and “Naardenensis” groups were mapped to a different reference genome. Therefore, the genomic coverage is lower and unevenly distributed among the scaffolds. Nevertheless, while most of the essential brewing genes have a corresponding homologous in the “Anomalus” group, similarities in “Naardenensis” and “Custersianus” group are hardly found, inferring the high divergence of this species and the reference group.

### Fermentation Performance and Production of Flavor Compounds in *Brettanomyces* Population

**FIGURE 5:**
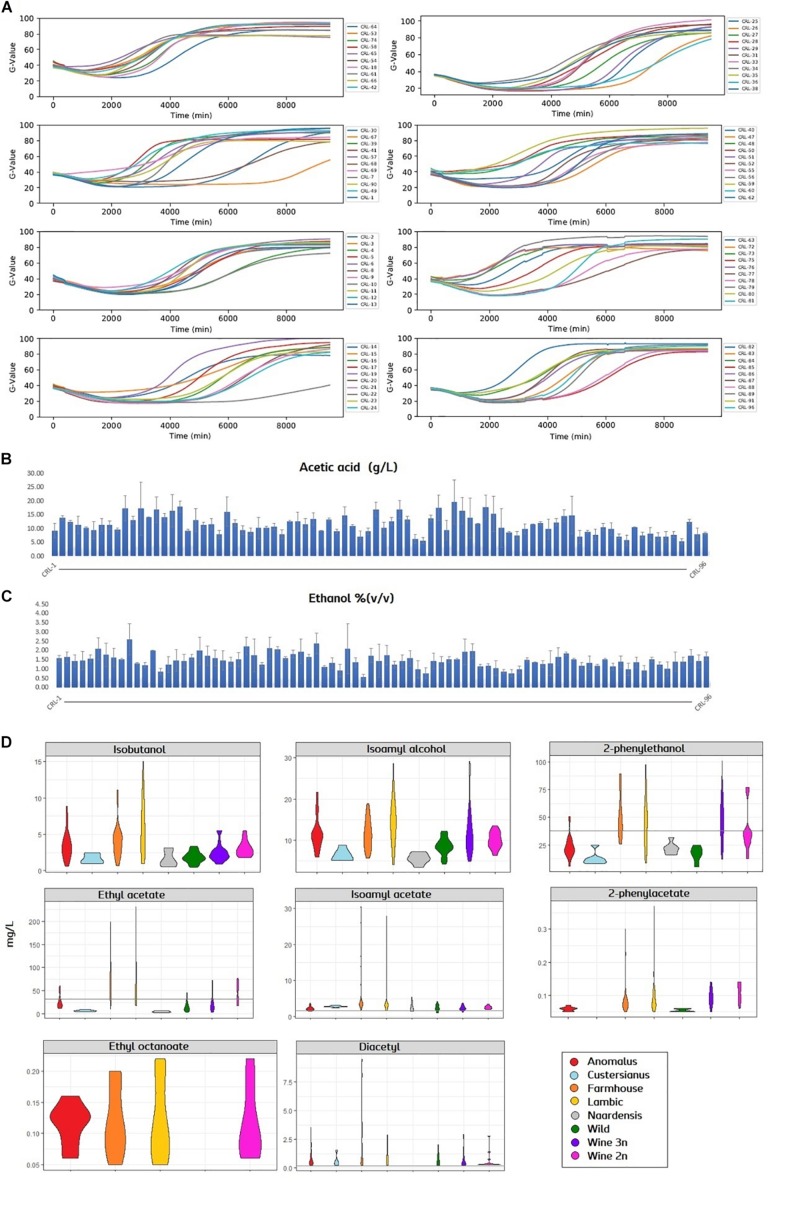
**(A)** Growth curves of *Brettanomyces* strain collection in pilsner wort, shown by means (*n* = 3). Growth-related values (G-value, *y*-axis) were extracted with the image analysis software (Enzyscreen). Fermentation time is indicated in minutes (*x*-axis). **(B,C)** Acetic acid and ethanol content of beers produced. Error bars show standard deviation (*n* = 3). **(D)** Violin plot for the main volatiles measured in beers. Strains are grouped according to its genetic cluster. Sensory detection threshold is indicated by a continuous line. When the line is not present, measured values are below detection threshold. Plots were obtained using ggplot2 package in R. Quantified values and statistical test can be found in Supplementary Table S2 and Statistical Test 1, respectively.

In order to assess the brewing potential of the *Brettanomyces* population, strains were inoculated into Pilsner wort, and fermentation was monitored in a 96-well plate in triplicates. Growth was quantified with a Growth-profiler^®^ Enzyscreen and growth kinetics curves were created (Figure 5A). Fermentations were stopped after 7 days when most of the strains had reached the stationary phase. Plates were centrifuged and the supernatant was collected for analysis. Acetic acid content was quantified varying from 5 to 20 g/L (Figure 5B and Supplementary Table S3). Also, ethanol content was measured from 0.5 to 2.5% v/v (Figure 5C and Supplementary Table S3). It should also be noted that for certain strains, the standard deviations are large among replicates (Supplementary Table S3). Despite plates being sealed with anaerobic lids, such high acetic acid/ethanol ratios indicate that oxygen was present during the wort fermentation (Aguilar Uscanga et al., 2003), and carbon utilization could have been poor in certain isolates. VOCs were quantified and a violin plot was produced to get an overview of the flavor distribution among genetic clusters (Figure 5D and Supplementary Table S2). In regard to fusel alcohols, the production of Isobutanol, 2-phenylethanol and isoamyl alcohol is generally higher in strains belonging to the genomic cluster (“Lambic,” “Farmhouse”), also “Anomalus.” Consistently, production of acetate esters is above average in such beer-related clusters. Certain strains such as CRL-21 and CRL-25 produce an outstanding amount of acetate esters, reaching levels above 26 mg/L of isoamyl acetate and 170 mg/L of ethyl acetate. Ethyl caprylate could only be measured in four groups and in small amounts. Diacetyl was detected in all samples except in “Naardenensis” group, most of it above sensory threshold of detection (0.05 mg/L).

### *Brettanomyces* Population Patterns in α- and β-Glucosidase Activity

**FIGURE 6:**
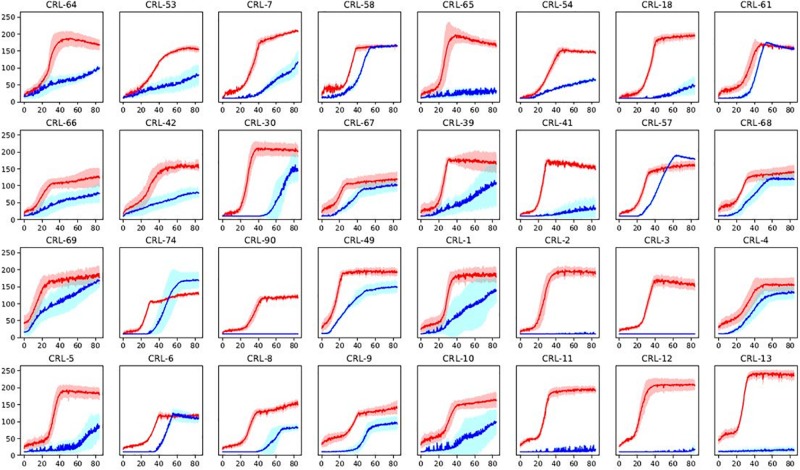
Sugar utilization of a representative part of *Brettanomyces* collection. Strains grown in media with glucose (red) or maltose (blue) as sole carbon source. Shaded curves are representing standard deviations between triplicates. *Y*-axis represents yeast growth quantified by Bvalue in Biolog. *X*-axis represents time in hours. Graphs of the full-collection can be found at Supplementary Figure S1. Statistical tests of the current data on maltose assimilation can be found in Supplementary Material.

Minimal media with a sole carbon source (glucose or maltose) was used in order to get an overview of α-glucosidase activity among the *Brettanomyces* collection. Growth was monitored by quantifying the colorimetric change in relation to NAD^+^ utilization, using Omnilog^®^ (BIOLOG). Kinetics of glucose and maltose utilization of the *Brettanomyces* collection is displayed in Figure 6. All the strains efficiently grow in the presence of glucose, confirming that glucose is the preferred substrate by *Brettanomyces* species (Blomqvist et al., 2010). However, when maltose is applied as a sole carbon source, a wide variability of responses is observed. Maltose is usually taken at lower rates than glucose, and several strains are not able to assimilate the sugar at all. These results indicate that alpha-glucosidase activity is strongly variable in between *Brettanomyces* species, and is not functional in some strains. Generally, there is a longer lag-phase when maltose is present, indicating that a regulatory mechanism is involved which can require adaptation time.

**FIGURE 7:**
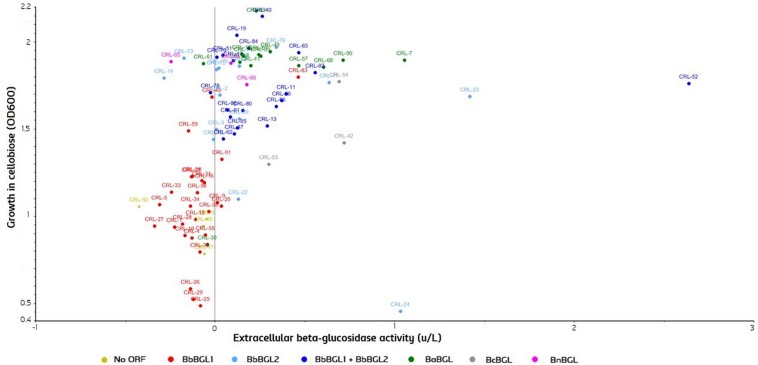
β-glucosidase activity in cultures grown on cellobiose as sole carbon source (OD600) and extracellular activity measured (units/L). Strains are grouped according to the identified ORF in the genome. Statistical tests of the current experimental data can be found in Supplementary Material.

To test β-glucosidase activity, strains were grown in YPC, using cellobiose as a sole carbon source, and OD600 was measured after 7 days. After that, yeast cells were removed by centrifugation, supernatant was collected, and conversion of beta substrates on the remaining media was measured. A Scatter plot comparing both activities was produced with the Unscrambler^®^CAMO (Figure 7). Strains are color-grouped according to the possession of genes. As expected, growth in cellobiose is strongly correlated with the beta-glucosidase activity in the extracellular fraction (Figure 7). Two β-glucosidases can be found in *B. bruxellensis*, and strains can have either none of them, just one, or both ORFs. Strains possessing no ORF or single *BbBGL1* do not grow efficiently on cellobiose, and consequently no β-glucosidase activity is observed. In contrast, strains possessing *BbBGL2* or both ORF display abundant growth on cellobiose and extracellular beta-glucosidase activity (Figure 7). These results clearly indicate that *BbBGL2* plays a major role in break-down beta glucosides, while the role of *BbBGL1* is minimum. As previously reported, the remaining *Brettanomyces* species (*anomalus*, *custersianus*, *naardenensis*) can grow efficiently on cellobiose and showed high β-glucosidase activity (Daenen et al., 2008a; Wu et al., 2014; Vervoort et al., 2016). β-glucosidase activity in the cellobiose-active strains is diverse as shown by *x*-axis in Figure 7. *B. bruxellensis* strain CRL-52 scored the highest conversion of β-glucosides measured in 2,56 units/L.

### Phenolic Off-Flavor Phenotype of *Brettanomyces* Population and Detection of POF-*Brettanomyces anomalus* CRL-90

**FIGURE 8:**
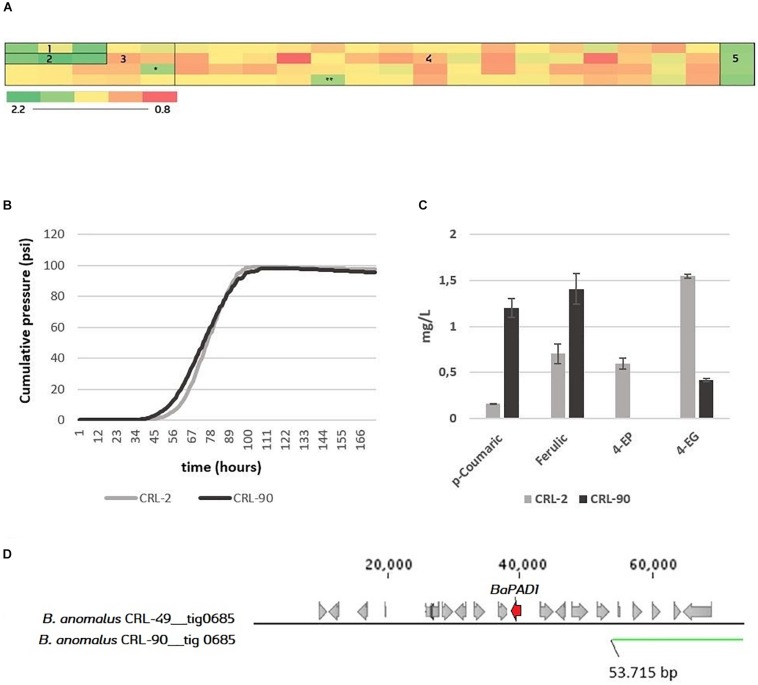
**(A)** Screening of the 84 *Brettanomyces* strains for uptake of ferulic acid. Heatmap displays averaged values of OD325 (*n* = 3). Standard deviations can be found at Supplementary Table S2. 1 = *B. naardenensis*; 2 = *B. custersianus;* 3 = *B. anomalus*; 4 = *B. bruxellensis*; 5 = Blanc; ^∗^ = CRL-90. ^∗∗^ = Low growth. For ferulic concentration, see Supplementary Table S3. **(B)** Pilsner wort fermentation of CRL-2 and CRL-90. Average values (*n* = 2). **(C)** Phenolic acids (p-coumaric, ferulic) and volatile phenols (4-ethylphenol, 4-ethylguaiacol) in mg/L at the end of fermentation. **(D)** Genomic setup of CRL-90 compared to the reference CRL-49. The figure has been produced with BLAST tool, comparing the contig nr. 685 where *DaPAD1* is present. In Supplementary Table S4, information about the presence of neighboring ORFs with its respective function is given.

The POF phenotype of the *Brettanomyces* population was investigated by measuring the uptake of the precursor ferulic acid. Strains were grown in YPD + 100 mg/L ferulic acid in triplicates in 96-deep well MTP format. After 7 days, the residual ferulic acid was detected by OD measurement at 325 nm. On one hand, results shown in Figure 8A indicate that both *B. custersianus* and *B. naardenensis* species are not able to assimilate ferulic acid. Only one *B. naardenensis* strain (CRL-65) seems to convert ferulic acid and be POF+. On the other hand, all strains of *B. anomalus* and *B. bruxellensis* are able to metabolize ferulic acid. Interestingly, one strain belonging to *B. anomalus* species (CRL-90) with a minimum conversion of ferulic acid was identified. To test CRL-90 POF phenotype further, wort fermentations in 250 mL glass bottles were performed, and the content of volatile phenols in the resulting beer was quantified (Figures 8B,C). While both 4-EP and 4-EG are detected in the control stain CRL-2, 4-EP is absent and a minimum amount of 4-EG is present after fermentation with CRL-90. The phenolic pathway intermediates 4-VP and 4-VG are below threshold in all fermentations. These results confirm the inability of CRL-90 to convert phenolic acids and therefore the first *Brettanomyces/Dekkera* species without POF up to date is reported. Such results are consistent with the genotypic data, as CRL-90 strain is missing *BaPAD1* gene responsible for decarboxylation of ferulic acid to 4-VG. When the corresponding scaffold is compared to CRL-49 reference genome, strain CRL-90 is missing the side region where *BaPAD1* is located (Figure 8D). Additionally, as loss-of-function mutations (such as frameshifts or premature stop codons) in *ScPAD1* and *ScFDC1* are commonly found (Gallone et al., 2016; Gonçalves et al., 2016; Preiss et al., 2017), we investigated their presence in *BbPAD1.* Nevertheless, amino acid sequences of *BbPAD1* resulted highly similar and none of the POF inactivation mechanisms previously mentioned were found.

### Genotype/Phenotype Correlation and Principal Component Analysis Based on Experimental Data

**FIGURE 9:**
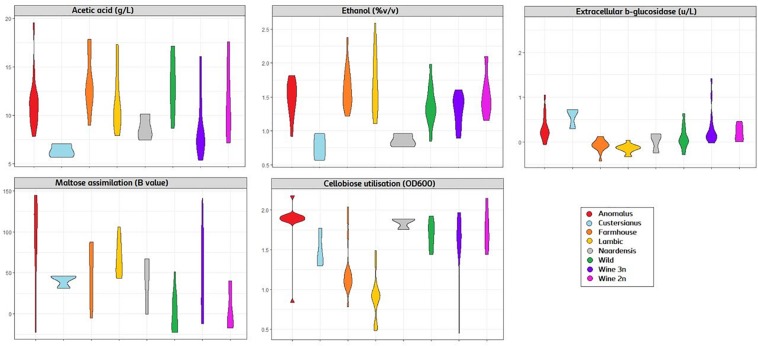
Violin plot with phenotypical measurements of *Brettanomyces* population. Strains are grouped according to the genomic cluster assigned in Figure 2.

**FIGURE 10:**
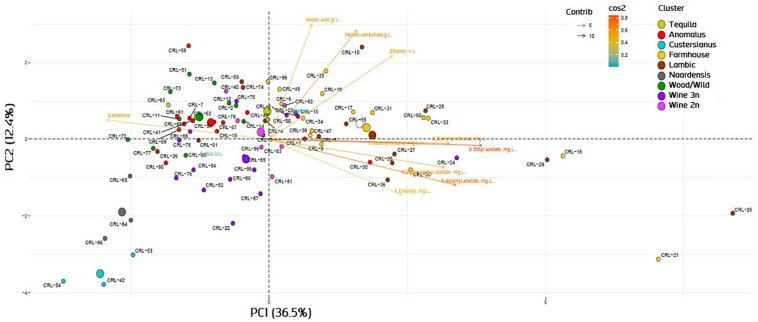
Bi-plot with principal component analysis variable loadings of *Brettanomyces* strains included in the study. Variable loadings are measured by cos2 function.

The genotype–phenotype correlation of the *Brettanomyces* population was investigated by grouping the strains according to its genomic cluster and plotting the phenotypical data generated (Figure 9). To summarize, *B. naardenensis* and *B. custersianus* confirm to be far distinct species, with specific properties as shown by low acetic acid and ethanol production along with ferulic acid consumption. With regard to alpha and beta-glucosidase activity, clear population trends are observed. “Anomalus” group displays powerful carbon assimilation, scoring high values in both cellobiose and maltose utilization. In regard to *B. bruxellensis* species, strains belonging to beer group (“Farmhouse,” “Lambic”) display efficient maltose utilization coupled to poor cellobiose assimilation. The opposite effect was observed in “Wine” and “Wild” isolates, as slow growth in maltose is linked to efficient growth in cellobiose. Our observations suggest that *B. bruxellensis* strains rely either on its α- or β-glucosidase activity to survive, and unnecessary traits for survival are lost over time. Furthermore, the population trends were visualized using a PCA of all previously quantified phenotypical parameters. A bi-plot was produced, with strains colored according to its genetic cluster (Figure 10). The weight of the variables into each PC is displayed by cos2 function with arrow length. Production of VOCs (as shown by concentrations) imposed a strong impact across PC1, explaining 36.5% of the variance. Variable loadings and correlation plot can be found in Supplementary Figures S2, S3. The *Brettanomyces* population distribution across the matrix in Figure 10 suggests that strain phenotypical properties can be predicted according to the genetic cluster they belong to.

## Discussion

In this study, we have aimed at providing a base to identify potential applications for *Brettanomyces* in brewing. A strain collection of *Brettanomyces* has been genotypically and phenotypically analyzed. Yeast strains have been clustered according to its predicted protein content, revealing specific genomic setups that enable survival in certain niches such as wine, beer, and wooden barrels. A large amount of functional genes have been identified, and the population has been sorted according to their presence or absence. The strains have also been tested for their brewing potential and a correlation with the genomic cluster has been carried out.

Throughout the study, several signs of artificial selection pressure have been observed. The short genetic divergence of beer isolates indicates that strains with similar characteristics have been selected for brewing. Such selection could have happened either in a selected manner, in Farmhouse brewing or in a spontaneous way in Lambic brewing (Spitaels et al., 2014; Colomer et al., 2019). This has led to a set of strains with a high degree of sugar attenuation and high ester production. In contrast, strains isolated from wine are largely divergent, whereas *Brettanomyces* species are considered spoilage yeasts. The pressure for natural adaptation is likely the cause of the genomic diversity observed. One example is the development of a triploid state for SO_2_ tolerance, pushing *Brettanomyces* wine-related population toward this characteristic (Borneman et al., 2014; Avramova et al., 2018a). Moreover, a singular cluster of strains has been characterized, containing strains isolated from bioethanol production plants and wooden barrels. This set of strains contain special properties, such as the inability of utilizing maltose as a substrate, balanced by the efficient degradation of cellobiose. In addition, *B. custersianus* and *B. naardenensis* species demonstrate interesting traits for brewing applications, like POF—phenotype and high β-glucosidase activity. In addition, *B. custersianus* displays a highly flocculent phenotype which is desired to ease brewing downstream processes. However, the low maltose assimilation and the production of several off-flavors such as H_2_S prevent these two species from being applied in brewing, at least in the short term. The potential application of *B. custersianus* and *B. naardenensis* for low alcoholic beers and secondary fermentation strains could be explored further.

Furthermore, *B. anomalus* has proven its potential for brewing, with an efficient metabolism of maltose, high β-glucosidase activity, and remarkable production of flavors (Figures 5D, 9). Additionally, one *B. anomalus* strain (CRL-90) was found to have a POF negative phenotype, related to the loss of the subtelomeric region containing *BaPAD1*. This confirms that BaPad1 protein is essential for the decarboxylation of ferulic acid (Godoy et al., 2014). CRL-90 is also the first strain within the *Brettanomyces* genus where such loss of POF function is described. Interestingly, the loss of the subtelomeric region containing *PAD1-FDC1* genetic cluster has been recently reported as a mechanism of POF inactivation in lager yeasts of the Saaz-type (Gallone et al., 2019; Langdon et al., 2019). Curiously, CRL-90 was also the only *Brettanomyces/Dekkera* strain with the inability of assimilating maltose, lacking also the maltose assimilation cluster (*MAL31*, *MAL11*, *IMA1*). This observation points toward a possible evolutionary correlation between the traits of maltose assimilation and POF production in *Brettanomyces* species (Shen et al., 2018).

In Farmhouse and Lambic brewing, *Brettanomyces* strains have been generally applied as “primary” fermentation strains, selecting toward an efficient maltose metabolism. Curiously, most of the *B. bruxellensis* beer isolates “Farmhouse” and “Lambic” score low on β-glucosidase activity and cannot metabolize cellobiose. This is due to the lack of *BbBGL2* gene which has shown to be essential on breaking-down beta-substrates (Figure 7). When possessing *BbBGL2 B. bruxellensis* can consume cellobiose, and have more probabilities to survive long term in wooden barrels or grape-peels (Crauwels et al., 2015a; Steensels et al., 2015). Therefore, *BbBGL2* could be considered as a potential gene target in production plants where *Brettanomyces* is not desired. As the concentration of α-glucosides in beer is substantially higher than the β-glucosides, most beer isolates have lost this non-essential ORF. In contrast, the absence of *BbBGL1* does not restrict the digestion of beta substrates, as shown by “Wine 3n” and “Wild” population. In terms of extracellular β-glucosidase activity, CRL-52 is outstanding from the rest of the population (Figure 7). Despite being a beer isolate, CRL-52 is genomically distinct from the main *B. bruxellensis* beer isolates, and could also be considered a potential common ancestor (Figure 2). CRL-52 was isolated in a South African “Bantu” brewery, where malted millet is used as raw material, requiring a different adaptation to the substrate composition for its survival. Moreover, CRL-52 is among the few strains capable to metabolize both maltose and cellobiose efficiently, confirming that unnecessary traits for survival (either α- or β-glucosidase activity) could have been eliminated in certain *Brettanomyces* populations over time.

Interestingly, the yeasts in the “Lambic” genetic cluster are missing nitrate assimilation ORFs, pointing toward nitrate uptake not being an essential requirement for survival in beer (Figure 4). Beer wort is widely known as an amino acid-rich media, therefore other nitrogen sources are available to use by this *Brettanomyces* population (Parente et al., 2018). Maltose assimilation in the studied *B. bruxellensis* isolates still remains ambiguous. Despite the maltose assimilation cluster (*MAL31*, *MAL11*, *IMA1*) is found in all the isolates, mostly beer and some wine isolates are capable of assimilating maltose, while “wild” strains are not. A gene coding for a maltase, *MAL12*, was initially thought to contribute to the population variance aiding the break-down of α-linked sugars (Crauwels et al., 2015b). Unexpectedly, most beer strains are missing this ORF and are still able to metabolize maltose, which was also seen before (Crauwels et al., 2017). While maltose assimilation has been extensively elucidated in brewer’s yeast, with complex regulatory pathways containing several repressors and regulators (Novak et al., 2004; Brickwedde et al., 2018), further research is needed to uncover such sugar metabolism in *Brettanomyces*. Despite population trends in regard to maltose assimilation phenotype are observed, the role of specific proteins is still uncertain. As in *S. cerevisiae*, maltose metabolism is probably determined by gene duplication and increase in copy number (Teste et al., 2010; Needleman and Michels, 2015).

With regard to flavor production, certain strains with high production of acetate esters have been identified (CRL-21 and CRL-25). Such phenotype could be due to a higher copy number of *ATF1* gene, as unique nucleotide sequences in any of these strains could not be identified. Production of fusel alcohols and acetate esters is generally higher in beer isolates, most likely due to efficient catabolism of amino acid precursors (valine, leucine, isoleucine, phenylalanine) (Hazelwood et al., 2008; Celińska et al., 2019). Furthermore, the off-flavor diacetyl was measured at extensive ranges, and no correlation could be defined. Most of the diacetyl-related *ILV* genes are located in centromeric-like regions and conserved in *B. bruxellensis* strains. As this off-flavor is linked to the fermentation–maturation stage of the beer, and its flavor detection threshold is remarkably low, it can become challenging to rush into certain conclusions for production and prevention of this metabolite (Dasari and Kölling, 2011; Pires et al., 2015).

## Conclusion

The present study reveals an extensive evaluation of the *Brettanomyces* population diversity worldwide, while also connects phenotypical traits to genotypes. A deep understanding of the huge range of properties *Brettanomyces* species are capable of offers a novel perspective into the phenotypic variants of this species. This opens opportunities for its industrial application, particularly in the field of beer brewing. This knowledge could be applied to satisfy the high demand for development of craft beers with novel flavors and beers with low- to non-alcohol content.

## Data Availability Statement

The genome sequence datasets generated in this study are available in NCBI BioProject number PRJNA592329.

## Author Contributions

MC conducted the experiments and wrote the manuscript. AC and RF supported bioinformatics analysis and figure creation. KO and LJ performed the quantification and data handling of volatile compounds. MC, NS, and JF designed the experiments. NS and JF contributed to supervision support. The manuscript was critically reviewed by all the authors and approved.

## Conflict of Interest

A patent application to protect some findings of this work has been filed by Carlsberg Breweries. AC, RF, KO, NS, and JF were employed by the company Carlsberg AS. LJ was employed by the company MS-Omics. The remaining author declares that the research was conducted in the absence of any commercial or financial relationships that could be construed as a potential conflict of interest.

## References

[B1] Aguilar Uscanga M. G. Délia M. L. Strehaiano P. (2003). *Brettanomyces bruxellensis*: effect of oxygen on growth and acetic acid production. *Appl. Microbiol. Biotechnol.* 61 157–162. 10.1007/s00253-002-1197-z 12655458

[B2] Aguilar-Uscanga M. G. Garcia-Alvarado Y. Gomez-Rodriguez J. Phister T. Delia M. L. Strehaiano P. (2011). Modelling the growth and ethanol production of *Brettanomyces bruxellensis* at different glucose concentrations. *Lett. Appl. Microbiol.* 53 141–149. 10.1111/j.1472-765X.2011.03081.x 21575020

[B3] Ames R. M. Rash B. M. Hentges K. E. Robertson D. L. Delneri D. Lovell S. C. (2010). Gene duplication and environmental adaptation within yeast populations. *Genome Biol. Evol.* 2 591–601. 10.1093/gbe/evq043 20660110 PMC2997561

[B4] Andersion R. A. Y. (2012). Brewery One Yeast or Two? Pure Yeast and Top Fermentation. *Brew. Hist.* 149 30–38.

[B5] Avramova M. Cibrario A. Peltier E. Coton M. Coton E. Schacherer J. (2018a). *Brettanomyces bruxellensis* population survey reveals a diploid-triploid complex structured according to substrate of isolation and geographical distribution. *Sci. Rep.* 8:4136. 10.1038/s41598-018-22580-7 29515178 PMC5841430

[B6] Avramova M. Vallet-Courbin A. Maupeu J. Masneuf-Pomarède I. Albertin W. (2018b). Molecular diagnosis of *Brettanomyces bruxellensis*’ sulfur dioxide sensitivity through genotype specific method. *Front. Microbiol.* 9:1260 10.3389/fmicb.2018.01260 PMC6004410 29942296

[B7] Bisson L. F. Karpel J. E. (2010). Genetics of Yeast Impacting Wine Quality. *Annu. Rev. Food Sci. Technol.* 1 139–162. 10.1146/annurev.food.080708.100734 22129333

[B8] Blomqvist J. Eberhard T. Schnürer J. Passoth V. (2010). Fermentation characteristics of *Dekkera bruxellensis* strains. *Appl. Microbiol. Biotechnol.* 87 1487–1497. 10.1007/s00253-010-2619-y 20437232

[B9] Borneman A. R. Zeppel R. Chambers P. J. Curtin C. D. (2014). Insights into the *Dekkera bruxellensis* genomic landscape: comparative genomics reveals variations in ploidy and nutrient utilisation potential amongst wine isolates. *PLoS Genet.* 10:e1004161. 10.1371/journal.pgen.1004161 24550744 PMC3923673

[B10] Brickwedde A. Brouwers N. van den Broek M. Gallego Murillo J. S. Fraiture J. L. Pronk J. T. (2018). Structural, physiological and regulatory analysis of maltose transporter genes in *Saccharomyces* eubayanus CBS 12357T. *Front. Microbiol.* 9:1786. 10.3389/fmicb.2018.01786 30147677 PMC6097016

[B11] Brown C. A. Murray A. W. Verstrepen K. J. (2010). Rapid expansion and functional divergence of subtelomeric gene families in yeasts. *Curr. Biol.* 20 895–903. 10.1016/j.cub.2010.04.027 20471265 PMC2877759

[B12] Callemien D. Collin S. (2010). Structure, organoleptic properties, quantification methods, and stability of phenolic compounds in beer-A review. *Food Rev. Int.* 26 1–84. 10.1080/87559120903157954

[B13] Celińska E. Borkowska M. Białas W. Kubiak M. Korpys P. Archacka M. (2019). Genetic engineering of Ehrlich pathway modulates production of higher alcohols in engineered *Yarrowia lipolytica*. *FEMS Yeast Res.* 19:foy122. 10.1093/femsyr/foy122 30452758

[B14] Chatonnet P. Dubourdie D. Boidron J.-N. Pons M. (1992). The origin of ethylphenols in wines. *J. Sci. Food Agric.* 60 165–178. 10.1002/jsfa.2740600205 18576949

[B15] Cibrario A. Miot-sertier C. Paulin M. Bullier B. Riquier L. Perello M. C. (2020). *Brettanomyces bruxellensis* phenotypic diversity, tolerance to wine stress and wine spoilage ability. *Food Microbiol.* 87:103379. 10.1016/j.fm.2019.103379 31948620

[B16] Claussen N. H. (1904). On a method for the application of Hansen’s pure yeast system in the manufacturing of well-conditioned english stock beers. *J. Inst. Brew.* 10 308–331. 10.1002/j.2050-0416.1904.tb04656.x

[B17] Claussen N. H. (1906). Manufacture of English beers or malt liquors. U.S. Patent No 1904208464A. Washington, DC: U.S. Patent and Trademark Office 10.1002/j.2050-0416.1904.tb04656.x

[B18] Colomer M. S. Funch B. Forster J. (2019). The raise of Brettanomyces yeast species for beer production. *Curr. Opin. Biotechnol.* 56 30–35. 10.1016/j.copbio.2018.07.009 30173102

[B19] Crauwels S. Steensels J. Aerts G. Willems K. A. Verstrepen K. J. Lievens B. (2015a). *Brettanomyces bruxellensis*, essential contributor in spontaneous beer fermentations providing novel opportunities for the brewing industry. *Brew. Sci.* 68 110–121.

[B20] Crauwels S. Van Assche A. de Jonge R. Borneman A. R. Verreth C. Troels P. (2015b). Comparative phenomics and targeted use of genomics reveals variation in carbon and nitrogen assimilation among different *Brettanomyces bruxellensis* strains. *Appl. Microbiol. Biotechnol.* 99 9123–9134. 10.1007/s00253-015-6769-9 26135985

[B21] Crauwels S. Van Opstaele F. Jaskula-Goiris B. Steensels J. Verreth C. Bosmans L. (2017). Fermentation assays reveal differences in sugar and (off-) flavor metabolism across different *Brettanomyces bruxellensis* strains. *FEMS Yeast Res.* 17:fow105. 10.1093/femsyr/fow105 27956491

[B22] Crauwels S. Zhu B. Steensels J. Busschaert P. De Samblanx G. Marchal K. (2014). Assessing genetic diversity among *Brettanomyces* yeasts by DNA fingerprinting and whole-genome sequencing. *Appl. Environ. Microbiol.* 80 4398–4413. 10.1128/AEM.00601-14 24814796 PMC4068659

[B23] Curtin C. D. Borneman A. R. Chambers P. J. Pretorius I. S. (2012). De-novo assembly and analysis of the heterozygous triploid genome of the wine spoilage yeast *Dekkera bruxellensis* AWRI1499. *PLoS One* 7:e33840. 10.1371/journal.pone.0033840 22470482 PMC3314683

[B24] Curtin C. D. Pretorius I. S. (2014). Genomic insights into the evolution of industrial yeast species *Brettanomyces bruxellensis*. *FEMS Yeast Res.* 14 997–1005. 10.1111/1567-1364.12198 25142832

[B25] Daenen L. Saison D. Sterckx F. Delvaux F. R. Verachtert H. Derdelinckx G. (2008a). Screening and evaluation of the glucoside hydrolase activity in *Saccharomyces* and *Brettanomyces* brewing yeasts. *J. Appl. Microbiol.* 104 478–488. 10.1111/j.1365-2672.2007.03566.x 17927762

[B26] Daenen L. Sterckx F. Delvaux F. R. Verachtert H. Derdelinckx G. (2008b). Evaluation of the glycoside hydrolase activity of a *Brettanomyces* strain on glycosides from sour cherry (*Prunus cerasus* L.) used in the production of special fruit beers. *FEMS Yeast Res.* 8 1103–1114. 10.1111/j.1567-1364.2008.00421.x 18673394

[B27] Daenen L. Vanderhaegen B. Verachtert H. Derdelinckx G. (2004). Flavour enhancement in beer by yeast beta-glucosidase activity. *Commun. Agric. Appl. Biol. Sci.* 69 73–76. 15560191

[B28] Dasari S. Kölling R. (2011). Cytosolic localization of acetohydroxyacid synthase Ilv2 and its impact on diacetyl formation during beer fermentation. *Appl. Environ. Microbiol.* 77 727–731. 10.1128/AEM.01579-10 21131528 PMC3028693

[B29] Deng X. Petitjean M. Teste M. A. Kooli W. Tranier S. François J. M. (2014). Similarities and differences in the biochemical and enzymological properties of the four isomaltases from *Saccharomyces cerevisiae*. *FEBS Open Bio* 4 200–212. 10.1016/j.fob.2014.02.004 24649402 PMC3953731

[B30] Dunn B. Richter C. Kvitek D. J. Pugh T. Sherlock G. (2012). Analysis of the *Saccharomyces cerevisiae* pan-genome reveals a pool of copy number variants distributed in diverse yeast strains from differing industrial environments. *Genome Res.* 22 908–924. 10.1101/gr.130310.111 22369888 PMC3337436

[B31] Duong C. T. Strack L. Futschik M. Katou Y. Nakao Y. Fujimura T. (2011). Identification of Sc-type ILV6 as a target to reduce diacetyl formation in lager brewers’ yeast. *Metab. Eng.* 13 638–647. 10.1016/j.ymben.2011.07.005 21824525

[B32] Duval E. H. Alves S. L. Dunn B. Sherlock G. Stambuk B. U. (2010). Microarray karyotyping of maltose-fermenting *Saccharomyces* yeasts with differing maltotriose utilization profiles reveals copy number variation in genes involved in maltose and maltotriose utilization. *J. Appl. Microbiol.* 109 248–259. 10.1111/j.1365-2672.2009.04656.x 20070441 PMC6394849

[B33] Emms D. M. Kelly S. (2015). OrthoFinder: solving fundamental biases in whole genome comparisons dramatically improves orthogroup inference accuracy. *Genome Biol.* 16:157. 10.1186/s13059-015-0721-2 26243257 PMC4531804

[B34] Emms D. M. Kelly S. (2017). STRIDE: species tree root inference from gene duplication events. *Mol. Biol. Evol.* 34 3267–3278. 10.1093/molbev/msx259 29029342 PMC5850722

[B35] Emms D. M. Kelly S. (2018). STAG: Species Tree Inference from All Genes. *bioRxiv* [Preprint]. 10.1101/267914 31875157

[B36] Emms D. M. Kelly S. (2019). OrthoFinder: phylogenetic orthology inference for comparative genomics. *Genome Biol.* 20:238. 10.1186/s13059-019-1832-y 31727128 PMC6857279

[B37] Fournier T. Gounot J.-S. Freel K. Cruaud C. Lemainque A. Aury J.-M. (2017). High-quality *de novo* genome assembly of the *Dekkera bruxellensis* yeast isolate using Nanopore MinION sequencing. *G3* 7 3243–3250. 10.1534/g3.117.300128 28983066 PMC5633375

[B38] Fujii T. Nagasawa N. Iwamatsu A. Bogaki T. Tamai Y. Hamachi M. (1994). Molecular cloning, sequence analysis, and expression of the yeast alcohol acetyltransferase gene. *Appl. Environ. Microbiol.* 60 2786–2792. 10.1128/aem.60.8.2786-2792.1994 8085822 PMC201724

[B39] Fujita S. I. Senda Y. Nakaguchi S. Hashimoto T. (2001). Multiplex PCR using internal transcribed spacer 1 and 2 regions for rapid detection and identification of yeast strains. *J. Clin. Microbiol.* 39 3617–3622. 10.1128/JCM.39.10.3617-3622.2001 11574582 PMC88398

[B40] Fukuda K. Kiyokawa Y. Yanagiuchi T. Wakai Y. Kitamoto K. Inoue Y. (2000). Purification and characterization of isoamyl acetate-hydrolyzing esterase encoded by the IAH1 gene of *Saccharomyces cerevisiae* from a recombinant *Escherichia coli*. *Appl. Microbiol. Biotechnol.* 53 596–600. 10.1007/s002530051662 10855721

[B41] Fukuda K. Yamamoto N. Kiyokawa Y. Yanagiuchi T. Wakai Y. Kitamoto K. (1998). Balance of activities of alcohol acetyltransferase and esterase in *Saccharomyces cerevisiae* is important for production of isoamyl acetate. *Appl. Environ. Microbiol.* 64 4076–4078. 10.1128/aem.64.10.4076-4078.1998 9758847 PMC106606

[B42] Gallone B. Steensels J. Mertens S. Dzialo M. C. Gordon J. L. Wauters R. (2019). Interspecific hybridization facilitates niche adaptation in beer yeast. *Nat. Ecol. Evol.* 3 1562–1575. 10.1038/s41559-019-0997-9 31636425

[B43] Gallone B. Steensels J. Prahl T. Soriaga L. Saels V. Herrera-Malaver B. (2016). Domestication and Divergence of *Saccharomyces cerevisiae* Beer Yeasts. *Cell* 166 1397–1410.e16. 10.1016/j.cell.2016.08.020 27610566 PMC5018251

[B44] Gibson B. Geertman J. M. A. Hittinger C. T. Krogerus K. Libkind D. Louis E. J. (2017). New yeasts-new brews: modern approaches to brewing yeast design and development. *FEMS Yeast Res.* 17:fox038. 10.1093/femsyr/fox038 28582493

[B45] Gilliland R. (1961). Brettanomyces: occurrence, characteristics and effects on beer flavor. *J. Inst. Brew.* 67:257 10.1002/j.2050-0416.1961.tb01791.x

[B46] Gjermansen C. Nilson T. Holmnberg S. (1988). Towards diacetyl-less brewer’s yeast. Influence of ILV2 and ILV5 mutations. *J. Basic Micribiol.* 28 175–183. 10.1002/jobm.3620280304 3057172

[B47] Godoy L. García V. Peña R. Martínez C. Ganga M. A. (2014). Identification of the *Dekkera bruxellensis* phenolic acid decarboxylase (PAD) gene responsible for wine spoilage. *Food Control* 45 81–86. 10.1016/j.foodcont.2014.03.041

[B48] Godoy L. Garrido D. Martínez C. Saavedra J. Combina M. Ganga M. A. (2009). Study of the coumarate decarboxylase and vinylphenol reductase activities of *Dekkera bruxellensis* (anamorph *Brettanomyces bruxellensis*) isolates. *Lett. Appl. Microbiol.* 48 452–457. 10.1111/j.1472-765X.2009.02556.x 19187489

[B49] Gonçalves M. Pontes A. Almeida P. Barbosa R. Serra M. Libkind D. (2016). Distinct domestication trajectories in top-fermenting beer yeasts and wine yeasts. *Curr. Biol.* 26 2750–2761. 10.1016/j.cub.2016.08.040 27720622

[B50] Gorter de Vries A. R. Pronk J. T. Daran J.-M. G. (2017). Industrial relevance of chromosomal copy number variation in *Saccharomyces* yeasts. *Appl. Environ. Microbiol.* 83 e3206–e3216. 10.1128/aem.03206-16 28341679 PMC5440705

[B51] Gounot J.-S. Neuveglise C. Freel K. C. Devillers H. Piskur J. Friedrich A. (2019). High complexity and degree of genetic variation in *Brettanomyces bruxellensis* population. *bioRxiv* [Preprint] 10.1101/826990 PMC7313668 32302403

[B52] Harari Y. Ram Y. Kupiec M. (2018). Frequent ploidy changes in growing yeast cultures. *Curr. Genet.* 64 1001–1004. 10.1007/s00294-018-0823-y 29525927

[B53] Hazelwood L. A. Daran J. M. Van Maris A. J. A. Pronk J. T. Dickinson J. R. (2008). The Ehrlich pathway for fusel alcohol production: a century of research on *Saccharomyces cerevisiae* metabolism. *Appl. Environ. Microbiol.* 74 2259–2266. 10.1128/AEM.02625-07 18281432 PMC2293160

[B54] Hellborg L. Piškur J. (2009). Complex nature of the genome in a wine spoilage yeast, *Dekkera bruxellensis*. *Eukaryot. Cell* 8 1739–1749. 10.1128/ec.00115-09 19717738 PMC2772400

[B55] Heresztyn T. (1986). Metabolism of volatile phenolic compounds from hydroxycinnamic acids by Brettanomyces yeast. *Arch. Microbiol.* 146 96–98. 10.1007/BF00690165

[B56] Hittinger C. T. Carroll S. B. (2007). Gene duplication and the adaptive evolution of a classic genetic switch. *Nature* 449 677–681. 10.1038/nature06151 17928853

[B57] Horák J. (2013). Regulations of sugar transporters: insights from yeast. *Curr. Genet.* 59 1–31. 10.1007/s00294-013-0388-8 23455612

[B58] Ihmels J. Bergmann S. Gerami-Nejad M. Yanai I. McClellan M. Berman J. (2005). Molecular biology: rewiring of the yeast transcriptional network through the evolution of motif usage. *Science* 309 938–940. 10.1126/science.1113833 16081737

[B59] Ishchuk O. P. Zeljko T. V. Schifferdecker A. J. Wisén S. M. Hagström Å. K. Rozpȩdowska E. (2016). Novel centromeric loci of the wine and beer yeast *Dekkera bruxellensis* CEN1 and CEN2. *PLoS One* 11:e0161741 10.1371/journal.pone.0161741 PMC4999066 27560164

[B60] Jespersen L. Cesar L. B. Meaden P. G. Jakobsen M. (1999). Multiple α-glucoside transporter genes in brewer’s yeast. *Appl. Environ. Microbiol.* 65 450–456. 10.1128/aem.65.2.450-456.1999 9925567 PMC91046

[B61] Koren S. Walenz B. P. Berlin K. Miller J. R. Bergman N. H. Phillippy A. M. (2017). Canu: Scalable and accurate long-read assembly via adaptive κ-mer weighting and repeat separation. *Genome Res.* 27 722–736. 10.1101/gr.215087.116 28298431 PMC5411767

[B62] Krogerus K. Gibson B. R. (2013). Influence of valine and other amino acids on total diacetyl and 2,3-pentanedione levels during fermentation of brewer’s wort. *Appl. Microbiol. Biotechnol.* 97 6919–6930. 10.1007/s00253-013-4955-1 23677441 PMC3708283

[B63] Kuo H. P. Wang R. Huang C. Y. Lai J. T. Lo Y. C. Huang S. T. (2018). Characterization of an extracellular β-glucosidase from *Dekkera bruxellensis* for resveratrol production. *J. Food Drug Anal.* 26 163–171. 10.1016/j.jfda.2016.12.016 29389552 PMC9332651

[B64] Kurtzman C. P. Fell J. W. Boekhout T. (eds) (2011). “The yeasts: a taxonomic study,” in *The Yeasts*, 5th Edn (Amsterdam: Elsevier), 1–3. 10.1017/CBO9781107415324.004

[B65] Langdon Q. K. Peris D. Baker E. C. P. Opulente D. A. Nguyen H. V. Bond U. (2019). Fermentation innovation through complex hybridization of wild and domesticated yeasts. *Nat. Ecol. Evol.* 3 1576–1586. 10.1038/s41559-019-0998-8 31636426 PMC7295394

[B66] Larroy C. Fernández M. R. González E. Parés X. Biosca J. A. (2002). Characterization of the *Saccharomyces cerevisiae* YMR318C (ADH6) gene product as a broad specificity NADPH-dependent alcohol dehydrogenase: relevance in aldehyde reduction. *Biochem. J.* 361 (Pt 1), 163–172. 10.1042/bj3610163 11742541 PMC1222291

[B67] Lebleux M. Abdo H. Coelho C. Basmaciyan L. Albertin W. Maupeu J. (2020). New advances on the *Brettanomyces bruxellensis* biofilm mode of life. *Int. J. Food Microbiol.* 318:108464. 10.1016/j.ijfoodmicro.2019.108464 31816527

[B68] Lentz M. Harris C. (2015). Analysis of growth inhibition and metabolism of hydroxycinnamic acids by brewing and spoilage strains of Brettanomyces yeast. *Foods* 4 581–593. 10.3390/foods4040581 28231223 PMC5224551

[B69] Li H. (2018). Minimap2: pairwise alignment for nucleotide sequences. *Bioinformatics* 34 3094–3100. 10.1093/bioinformatics/bty191 29750242 PMC6137996

[B70] Li H. Handsaker B. Wysoker A. Fennell T. Ruan J. Homer N. (2009). The Sequence Alignment/Map format and SAMtools. *Bioinformatics* 25 2078–2079. 10.1093/bioinformatics/btp352 19505943 PMC2723002

[B71] Libkind D. Hittinger C. T. Valerio E. Goncalves C. Dover J. Johnston M. (2011). Microbe domestication and the identification of the wild genetic stock of lager-brewing yeast. *Proc. Natl. Acad. Sci. U.S.A.* 108 14539–14544. 10.1073/pnas.1105430108 21873232 PMC3167505

[B72] Miklenić M. Žunar B. Štafa A. Svetec I. K. (2015). Improved electroporation procedure for genetic transformation of dekkera/*brettanomyces bruxellensis*. *FEMS Yeast Res.* 15:fov096 10.1093/femsyr/fov096 26542709

[B73] Mukai N. Masaki K. Fujii T. Kawamukai M. Iefuji H. (2010). PAD1 and FDC1 are essential for the decarboxylation of phenylacrylic acids in *Saccharomyces cerevisiae*. *J. Biosci. Bioeng.* 109 564–569. 10.1016/j.jbiosc.2009.11.011 20471595

[B74] Naumoff D. G. Naumov G. I. (2010). Discovery of a novel family of α-glucosidase IMA genes in yeast *Saccharomyces cerevisiae*. *Biochem. Biophys.* 432 114–116. 10.1134/s1607672910030051 20886742

[B75] Needleman R. B. Kaback D. B. Dubin R. A. Perkins E. L. Rosenberg N. G. Sutherland K. A. (1984). MAL6 of *Saccharomyces*: a complex genetic locus containing three genes required for maltose fermentation. *Proc. Natl. Acad. Sci. U.S.A.* 81 2811–2815. 10.1073/pnas.81.9.2811 6371820 PMC345160

[B76] Needleman R. B. Michels C. (2015). Repeated family of genes controlling maltose fermentation in *Saccharomyces carlsbergensis*. *Mol. Cell. Biol.* 3 796–802. 10.1128/mcb.3.5.796 6346055 PMC368602

[B77] Novak S. Zechner-Krpan V. Marić V. (2004). Regulation of maltose transport and metabolism in *Saccharomyces cerevisiae*. *Food Technol. Biotechnol.* 42 213–218.

[B78] Olsen R. A. Bunikis I. Tiukova I. Holmberg K. Lötstedt B. Pettersson O. V. (2015). De novo assembly of *Dekkera bruxellensis*: a multi technology approach using short and long-read sequencing and optical mapping. *GigaScience* 4:56. 10.1186/s13742-015-0094-1 26617983 PMC4661999

[B79] Parente D. C. Cajueiro D. B. B. Moreno I. C. P. Leite F. C. B. De Barros Pita W. De Morais M. A. (2018). On the catabolism of amino acids in the yeast *Dekkera bruxellensis* and the implications for industrial fermentation processes. *Yeast* 35 299–309. 10.1002/yea.3290 29065215

[B80] Peter J. De Chiara M. Friedrich A. Yue J.-X. Pflieger D. Bergström A. (2018). Genome evolution across 1,011 *Saccharomyces cerevisiae* isolates. *Nature* 556 339–344. 10.1038/s41586-018-0030-5 29643504 PMC6784862

[B81] Pinu F. R. Villas-Boas S. G. (2017). Rapid quantification of major volatile metabolites in fermented food and beverages using gas chromatography-mass spectrometry. *Metabolites* 7:37. 10.3390/metabo7030037 28933773 PMC5618322

[B82] Pires E. J. Teixeira J. A. Brányik T. Brandão T. Vicente A. A. (2015). Continuous beer fermentation - diacetyl as a villain. *J. Inst. Brew.* 121 55–61. 10.1002/jib.205

[B83] Piškur J. Ling Z. Marcet-Houben M. Ishchuk O. P. Aerts A. LaButti K. (2012). The genome of wine yeast *Dekkera bruxellensis* provides a tool to explore its food-related properties. *Int. J. Food Microbiol.* 157 202–209. 10.1016/j.ijfoodmicro.2012.05.008 22663979

[B84] Preiss R. Tyrawa C. Van Der Merwe G. Biology C. Laboratories E. Author C. (2017). Traditional norwegian Kveik Yeasts: underexplored domesticated *Saccharomyces cerevisiae* Yeasts. *BioRxiv* [Preprint]. 10.1101/194969

[B85] QIAGEN (2016). *CLC Genomics Workbench User Manual.* Hilden: QIAGEN.

[B86] Roach M. J. Borneman A. R. (2020). New genome assemblies reveal patterns of domestication and adaptation across Brettanomyces (Dekkera) species. *BMC Genomics* 21:194 10.1186/s12864-020-6595-z PMC7052964 32122298

[B87] Romano D. Valdetara F. Zambelli P. Galafassi S. De Vitis V. Molinari F. (2017). Cloning the putative gene of vinyl phenol reductase of *Dekkera bruxellensis* in *Saccharomyces cerevisiae*. *Food Microbiol.* 63 92–100. 10.1016/j.fm.2016.11.003 28040186

[B88] Rozpedowska E. Hellborg L. Ishchuk O. P. Orhan F. Galafassi S. Merico A. (2011). Parallel evolution of the make-accumulate-consume strategy in *Saccharomyces* and Dekkera yeasts. *Nat. Commun.* 2:302. 10.1038/ncomms1305 21556056 PMC3112538

[B89] Saerens S. M. G. Verstrepen K. J. Van Laere S. D. M. Voet A. R. D. Van Dijck P. Delvaux F. R. (2006). The *Saccharomyces cerevisiae* EHT1 and EEB1 genes encode novel enzymes with medium-chain fatty acid ethyl ester synthesis and hydrolysis capacity. *J. Biol. Chem.* 281 4446–4456. 10.1074/jbc.M512028200 16361250

[B90] Sanchez R. G. Solodovnikova N. Wendland J. (2012). Breeding of lager yeast with *Saccharomyces cerevisiae* improves stress resistance and fermentation performance. *Yeast* 29 343–355. 10.1002/yea.2914 22887121

[B91] Schifferdecker A. J. Dashko S. Ishchuk O. P. Piškur J. (2014). The wine and beer yeast *Dekkera bruxellensis*. *Yeast* 31 323–332. 10.1002/yea.3023 24932634 PMC4257070

[B92] Schifferdecker A. J. Siurkus J. Andersen M. R. Joerck-Ramberg D. Ling Z. Zhou N. (2016). Alcohol dehydrogenase gene ADH3 activates glucose alcoholic fermentation in genetically engineered *Dekkera bruxellensis* yeast. *Appl. Microbiol. Biotechnol.* 100 3219–3231. 10.1007/s00253-015-7266-x 26743658 PMC4786601

[B93] Schiønning H. (1908). On Torula in English Beer Manufacture. *Journal of the Institute of Brewing* 15 2–35. 10.1002/j.2050-0416.1909.tb02237.x

[B94] Shen X. Opulente D. A. Kominek J. Kurtzman C. P. Hittinger C. T. Rokas A. (2018). Tempo and mode of genome evolution in the budding yeast subphylum. *Cell* 175 1533–1545.e20. 10.1016/j.cell.2018.10.023 30415838 PMC6291210

[B95] Smith B. D. Divol B. (2016). *Brettanomyces bruxellensis*, a survivalist prepared for the wine apocalypse and other beverages. *Food Microbiol.* 59 161–175. 10.1016/j.fm.2016.06.008 27375257

[B96] Smith B. D. Divol B. (2018). The carbon consumption pattern of the spoilage yeast *Brettanomyces bruxellensis* in synthetic wine-like medium. *Food Microbiol.* 73 39–48. 10.1016/j.fm.2017.12.011 29526225

[B97] Smith M. T. (2011). “Dekkera van der Walt (1964),” in *The Yeasts*, Vol. 2 eds Kurtzman C. P. Fell J. W. (Amsterdam: Elsevier), 373–377. 10.1016/B978-0-444-52149-1.00025-2

[B98] Spitaels F. Wieme A. D. Janssens M. Aerts M. Daniel H. M. Van Landschoot A. (2014). The microbial diversity of traditional spontaneously fermented lambic beer. *PLoS One* 9:e95384. 10.1371/journal.pone.0095384 24748344 PMC3991685

[B99] Stanke M. Keller O. Gunduz I. Hayes A. Waack S. Morgenstern B. (2006). AUGUSTUS: A b initio prediction of alternative transcripts. *Nucleic Acids Res.* 34 W435–W439. 10.1093/nar/gkl200 16845043 PMC1538822

[B100] Steensels J. (2017). Rapid Screening Method for Phenolic Off-Flavor (POF) Production in Yeast. *J. Am. Soc. Brew. Chem.* 75 318–323. 10.1094/ASBCJ-2017-4142-01

[B101] Steensels J. Daenen L. Malcorps P. Derdelinckx G. Verachtert H. Verstrepen K. J. (2015). Brettanomyces yeasts - From spoilage organisms to valuable contributors to industrial fermentations. *Int. J. Food Microbiol.* 206 24–38. 10.1016/j.ijfoodmicro.2015.04.005 25916511

[B102] Steensels J. Meersman E. Snoek T. Saels V. Verstrepen K. J. (2014). Large-scale selection and breeding to generate industrial yeasts with superior aroma production. *Appl. Environ. Microbiol.* 80 6965–6975. 10.1128/aem.02235-14 25192996 PMC4249010

[B103] Teste M. A. Marie François J. Parrou J. L. (2010). Characterization of a new multigene family encoding isomaltases in the yeast *Saccharomyces cerevisiae*, the IMA family. *J. Biol. Chem.* 285 26815–26824. 10.1074/jbc.M110.145946 20562106 PMC2930680

[B104] Teunissen A. W. R. H. Steensma H. Y. (1995). The dominant flocculation genes of *Saccharomyces cerevisiae* constitute a new subtelomeric gene family. *Yeast* 11 1001–1013. 10.1002/yea.320111102 7502576

[B105] Tiukova I. A. Jiang H. Dainat J. Hoeppner M. P. Lantz H. Piskur J. (2019). Assembly and analysis of the genome sequence of the yeast *Brettanomyces naardenensis* CBS 7540. *Microorganisms* 7:489. 10.3390/microorganisms7110489 31717754 PMC6921048

[B106] Tkach J. M. Yimit A. Lee A. Y. Riffle M. Costanzo M. Jaschob D. (2012). Dissecting DNA damage response pathways by analysing protein localization and abundance changes during DNA replication stress. *Nat. Cell Biol.* 14 966–976. 10.1038/ncb2549 22842922 PMC3434236

[B107] Van Mulders S. E. Christianen E. Saerens S. M. G. Daenen L. Verbelen P. J. Willaert R. (2009). Phenotypic diversity of Flo protein family-mediated adhesion in *Saccharomyces cerevisiae*. *FEMS Yeast Res.* 9 178–190. 10.1111/j.1567-1364.2008.00462.x 19087208

[B108] Vanoni M. Sollitti P. Goldenthal M. Marmur J. (1989). Structure and regulation of the multigene family controlling maltose fermentation in budding yeast. *Prog. Nucleic Acid Res. Mol. Biol.* 37 281–322. 10.1016/S0079-6603(08)60701-1 2672110

[B109] Varela C. Lleixà J. Curtin C. Borneman A. (2018). Development of a genetic transformation toolkit for *Brettanomyces bruxellensis*. *FEMS Yeast Res.* 18:foy070. 10.1093/femsyr/foy070 29982550

[B110] Vervoort Y. Herrera-Malaver B. Mertens S. Guadalupe Medina V. Duitama J. Michiels L. (2016). Characterization of the recombinant Brettanomyces anomalus β-glucosidase and its potential for bioflavouring. *J. Appl. Microbiol.* 121 721–733. 10.1111/jam.13200 27277532 PMC6680314

[B111] Woolfit M. Rozpȩdowska E. Piškur J. Wolfe K. H. (2007). Genome survey sequencing of the wine spoilage yeast Dekkera (Brettanomyces) bruxellensis. *Eukaryot. Cell* 6 721–733. 10.1128/EC.00338-06 17277171 PMC1865652

[B112] Wu P. Zhao X. Pan S. (2014). Intraspecific protoplast fusion of Brettanomyces anomalus for improved production of an extracellular β-glucosidase. *Biotechnol. Biotechnol. Equip.* 28 878–881. 10.1080/13102818.2014.955290 26019572 PMC4434049

