# Supplementary material for: Assessing Population Diversity of Brettanomyces Yeast Species and Identification of Strains for Brewing Applications
Source: Front Microbiol. 2020 Apr 9;11:637. doi: 10.3389/fmicb.2020.00637 (PMC7177047; doi:10.3389/fmicb.2020.00637)
Supplement: Supplementary file 1 [file Data_Sheet_1.docx]

**Supplementary Material: Analysis of population diversity of *Brettanomyces* yeast species and identification of strains for brewing application**

| Gene name | Description | Brewing relevance |
| --- | --- | --- |
| ATF1 | Alcohol acetyltransferase | Ester production |
| BAT2 | Branched-chain-amino-acid aminotransferase | Ester production |
| ARO3 | 3-deoxy-D-arabino-heptulosonate-7-phosphate (DAHP) synthase | Ester production |
| ARO8 | Aromatic aminotransferase I | Ester production |
| EHT1 | Medium-chain fatty acid ethyl ester synthase | Ester production |
| ARO1 | Pentafunctional AROM polypeptide | Ester production |
| ARO80 | Zinc finger transcriptional activator of the Zn2Cys6 family | Ester production |
| YAT2 | Carnitine acetyltransferase | Ester production |
| ARO10 | Phenylpyruvate decarboxylase | Ester production |
| GCN4 | Transcriptional activator of amino acid biosynthetic genes | Ester production |
| ARO4 | 3-deoxy-D-arabino-heptulosonate-7-phosphate (DAHP) synthase | Ester production |
| IAH1 | Isoamyl acetate-hydrolyzing esterase | Ester production |
| ILV5 | Acetohydroxyacid reductoisomerase and mtDNA binding protein | Diacetyl production |
| ILV6 | Regulatory subunit of acetolactate synthase | Diacetyl production |
| ILV2 | Acetolactate synthase | Diacetyl production |
| ILV3 | Dihydroxyacid dehydratase | Diacetyl production |
| BDH1 | Butanediol dehydrogenase | Diacetyl production |
| PAD1 | Phenylacrylic acid decarboxylase | Phenolic off-flavor |
| SODC | Superoxide dismutase | Phenolic off-flavor |
| MAL31 | Maltose permease, transporter | Maltose assimilation |
| MAL11 (AGT1) | High-affinity maltose transporter | Maltose assimilation |
| IMA1 | Major isomaltase | Maltose assimilation |
| MAL12 | Maltase | Maltose assimilation |
| MAL61 | High-affinity maltose transporter | Maltose assimilation |
| MPH2 | Maltose permease | Maltose assimilation |
| MPH3 | Maltose permease | Maltose assimilation |
| HXT14 | Hexose transporter | Maltose assimilation |
| LACP | Lactose transporter | Maltose assimilation |
| EXG1 | Beta-glucosidase | Beta-glucoside release |
| EXG2 | Beta-glucosidase | Beta-glucoside release |
| BGL1 | Beta-glucosidase | Beta-glucoside release |
| BGL2 | Beta-glucosidase | Beta-glucoside release |
| ADH2 | Glucose-repressible alcohol dehydrogenase II | Ethanol production |
| ADH3 | Mitochondrial alcohol dehydrogenase isozyme III | Ethanol production |
| ADH6 | Medium chain alcohol dehydrogenase | Ethanol production |
| ADHC | Alcohol dehydrogenase | Ethanol production |
| FLO11 | GPI-anchored cell surface glycoprotein (flocculin) | Flocculation |
| FLO9 | Lectin-like protein with similarity to Flo1p | Flocculation |
| FLO8 | Transcription factor; required for flocculation | Flocculation |
| FLO10 | Member of the FLO family of cell wall flocculation proteins | Flocculation |
| FLO1 | Lectin-like protein involved in flocculation | Flocculation |
| MET5 | Sulfite reductase beta subunit | Sulphite tolerance |
| SSU1 | Plasma membrane sulfite pump involved in sulfite metabolism | Sulphite tolerance |
| HYE2 | NADPH oxidoreductase containing flavin mononucleotide (FMN) | Monoterpene alcohol conversion |
| HYE3 | NADPH oxidoreductase containing flavin mononucleotide (FMN) | Monoterpene alcohol conversion |
| YNT1 | Nitrate transporter | Nitrate assimilation |
| YNI1 | Nitrite reductase | Nitrate assimilation |
| YNR1 | Nitrate reductase | Nitrate assimilation |
| CEN1 | Centromeric loci | Genome rearrangement |
| CEN2 | Centromeric loci | Genome rearrangement |

Table S1. Predicted brewing-relevant genes identified in the reference genome of *B. bruxellensis* *UMY321* (Fournier et al., 2017).

Table S2: Volatile compounds measured with GC/MS in wort fermentations. STDV, n=3.

|  |  | **"Diacetyl (mg/L)"** | | **"Isobutanol (mg/L)"** | | **"Isoamyl acetate (mg/L)"** | | **"Ethyl octanoate (mg/L)"** | | **"2-phenylethyl acetate (mg/L)"** | | **"Isoamyl alcohol (mg/L)"** | | **"2-phenyl ethanol (mg/L)"** | | **"Ethyl acetate (mg/L)"** | |
| --- | --- | --- | --- | --- | --- | --- | --- | --- | --- | --- | --- | --- | --- | --- | --- | --- | --- |
|  |  |  |  |  |  |  |  |  |  |  |  |  |  |  |  |  |  |
|  | **Cluster** | **Average** | **STDV** | **Average** | **STDV** | **Average** | **STDV** | **Average** | **STDV** | **Average** | **STDV** | **Average** | **STDV** | **Average** | **STDV** | **Average** | **STDV** |
| CRL-6 | - | 0.35 | 0.11 | 3.25 | 0.57 | 2.31 | 0.01 | n.d. | n.d. | 0.07 | 0.01 | 10.58 | 0.47 | 44.77 | 0.74 | 25.07 | 3.85 |
| CRL-52 | - | 0.27 | n.d. | 3.75 | 0.61 | 1.85 | 0.12 | n.d. | n.d. | 0.06 | 0.01 | 14.33 | 1.22 | 32.89 | 4.84 | 33.46 | 9.52 |
| CRL-60 | - | 0.42 | 0.09 | 7.00 | 0.57 | 3.13 | 0.02 | n.d. | n.d. | 0.11 | 0.02 | 12.76 | 0.56 | 23.70 | 9.84 | 44.86 | 16.28 |
| CRL-63 | - | 0.29 | n.d. | 1.26 | 0.32 | 1.46 | 0.09 | n.d. | n.d. | 0.09 | 0.00 | 6.14 | 1.39 | 24.76 | 8.09 | 10.93 | 1.44 |
| CRL-7 | anomalus | 1.63 | 0.49 | 1.46 | 0.01 | 2.06 | 0.02 | n.d. | n.d. | 0.05 | 0.00 | 6.50 | 0.75 | 17.03 | 0.18 | 21.11 | 11.09 |
| CRL-18 | anomalus | 0.88 | 0.29 | 2.76 | 0.84 | 2.38 | 0.18 | n.d. | n.d. | 0.06 | 0.00 | 10.35 | 2.41 | 20.57 | 1.65 | 43.92 | 25.03 |
| CRL-30 | anomalus | 2.10 | 0.62 | 7.61 | 1.00 | 3.50 | 0.22 | 0.09 | n.d. | 0.07 | 0.00 | 19.39 | 2.13 | 49.46 | 1.62 | 43.84 | 7.82 |
| CRL-39 | anomalus | 0.44 | 0.02 | 2.07 | 0.49 | 2.24 | 0.14 | n.d. | n.d. | 0.06 | 0.00 | 7.84 | 1.31 | 20.44 | 1.49 | 17.71 | 2.85 |
| CRL-41 | anomalus | 0.34 | 0.07 | 3.34 | 0.18 | 1.86 | 0.38 | n.d. | n.d. | 0.06 | 0.01 | 11.25 | 0.41 | 21.73 | 9.06 | 16.59 | 3.94 |
| CRL-49 | anomalus | 0.91 | 0.13 | 3.29 | 0.88 | 2.64 | 0.27 | 0.12 | 0.02 | 0.06 | 0.01 | 10.64 | 1.81 | 31.55 | 1.82 | 47.31 | 18.12 |
| CRL-57 | anomalus | 0.72 | 0.46 | 5.43 | 2.97 | 1.52 | 0.12 | 0.13 | n.d. | n.d. | n.d. | 13.84 | 3.19 | 16.69 | 6.13 | 24.57 | 5.77 |
| CRL-58 | anomalus | 0.37 | 0.06 | 0.76 | 0.15 | 1.87 | 0.01 | n.d. | n.d. | 0.05 | n.d. | 6.62 | 0.85 | 19.49 | 0.42 | 17.83 | 4.73 |
| CRL-61 | anomalus | 0.43 | 0.11 | 1.54 | 0.40 | 1.99 | 0.27 | n.d. | n.d. | n.d. | n.d. | 10.05 | 1.02 | 6.02 | 0.62 | 21.12 | 5.68 |
| CRL-67 | anomalus | 0.49 | 0.05 | 4.36 | 0.25 | 1.88 | 0.25 | n.d. | n.d. | 0.06 | 0.01 | 18.11 | 2.92 | 23.83 | 7.47 | 20.39 | 6.86 |
| CRL-68 | anomalus | 0.82 | 0.42 | 2.69 | 0.52 | 1.84 | 0.10 | n.d. | n.d. | 0.05 | 0.00 | 11.25 | 1.70 | 18.46 | 1.36 | 27.33 | 11.78 |
| CRL-69 | anomalus | 0.40 | 0.05 | 2.94 | 0.36 | 2.77 | 0.33 | n.d. | n.d. | 0.06 | 0.00 | 9.58 | 1.10 | 25.49 | 4.61 | 24.27 | 3.20 |
| CRL-74 | anomalus | 1.85 | 1.48 | 4.22 | 2.41 | 2.37 | 0.12 | 0.06 | n.d. | 0.05 | 0.01 | 10.12 | 1.33 | 25.57 | 2.71 | 19.57 | 2.20 |
| CRL-90 | anomalus | 0.28 | 0.01 | 4.81 | 0.05 | 1.97 | 0.06 | n.d. | n.d. | n.d. | n.d. | 12.15 | 0.28 | 19.15 | 4.48 | 17.28 | 5.36 |
| CRL-42 | custersianus | 1.03 | 0.75 | 1.88 | 0.82 | 2.78 | 0.02 | n.d. | n.d. | 0.06 | n.d. | 6.87 | 0.27 | 8.91 | 1.30 | 7.85 | 1.94 |
| CRL-53 | custersianus | 0.60 | n.d. | 1.71 | 1.03 | 2.64 | 0.40 | n.d. | n.d. | 0.05 | n.d. | 6.97 | 2.60 | 18.56 | 8.97 | 6.03 | 1.57 |
| CRL-54 | custersianus | 0.28 | 0.01 | 1.35 | 0.47 | 2.90 | 0.25 | n.d. | n.d. | n.d. | n.d. | 5.21 | 0.06 | 11.14 | 2.54 | 6.80 | 1.51 |
| CRL-9 | farmhouse | 0.89 | 0.52 | 3.99 | 1.83 | 3.40 | 0.61 | 0.06 | n.d. | 0.08 | 0.02 | 11.81 | 4.10 | 71.95 | 24.55 | 29.91 | 16.13 |
| CRL-16 | farmhouse | 0.99 | 0.44 | 6.91 | 2.89 | 13.14 | 3.98 | 0.07 | 0.03 | 0.26 | 0.04 | 10.31 | 1.67 | 75.73 | 14.15 | 154.61 | 23.07 |
| CRL-17 | farmhouse | 0.42 | 0.04 | 0.99 | 0.38 | 2.02 | 0.28 | n.d. | n.d. | 0.06 | 0.01 | 6.36 | 0.97 | 28.34 | 0.82 | 41.72 | 15.54 |
| CRL-19 | farmhouse | 0.41 | 0.10 | 3.64 | 0.96 | 3.47 | 0.49 | 0.12 | 0.01 | 0.08 | 0.01 | 12.33 | 3.50 | 52.54 | 15.94 | 98.27 | 15.31 |
| CRL-20 | farmhouse | 3.34 | 0.44 | 4.93 | 0.19 | 4.95 | 0.13 | n.d. | n.d. | 0.08 | 0.00 | 15.88 | 0.97 | 52.61 | 3.59 | 76.67 | 1.30 |
| CRL-21 | farmhouse | 8.79 | 0.98 | 10.35 | 1.07 | 28.28 | 3.10 | 0.19 | 0.01 | 0.18 | 0.08 | 18.19 | 0.95 | 81.10 | 10.97 | 178.86 | 26.95 |
| CRL-31 | farmhouse | 1.00 | 0.49 | 4.03 | 1.80 | 3.44 | 0.68 | 0.20 | n.d. | 0.09 | 0.02 | 12.06 | 4.19 | 63.47 | 22.73 | 89.26 | 23.04 |
| CRL-33 | farmhouse | 1.39 | 0.10 | 5.71 | 1.29 | 4.40 | 0.00 | 0.09 | 0.01 | 0.10 | 0.01 | 16.42 | 2.00 | 82.51 | 5.19 | 77.63 | 17.92 |
| CRL-34 | farmhouse | 0.56 | 0.13 | 4.95 | 0.78 | 3.27 | 0.20 | n.d. | n.d. | 0.07 | 0.01 | 12.35 | 0.36 | 42.15 | 1.68 | 78.39 | 0.27 |
| CRL-35 | farmhouse | 0.32 | 0.01 | 3.14 | 0.48 | 2.94 | 0.13 | n.d. | n.d. | 0.06 | 0.01 | 10.51 | 0.49 | 34.80 | 1.44 | 73.05 | 18.06 |
| CRL-38 | farmhouse | 1.55 | 0.19 | 3.99 | 0.65 | 3.70 | 0.16 | n.d. | n.d. | 0.06 | 0.01 | 11.05 | 0.97 | 47.44 | 4.33 | 56.54 | 10.52 |
| CRL-47 | farmhouse | 0.31 | 0.03 | 4.07 | 0.81 | 3.95 | 0.30 | n.d. | n.d. | 0.07 | 0.01 | 11.81 | 2.39 | 33.63 | 7.00 | 59.01 | 11.17 |
| CRL-48 | farmhouse | 0.34 | 0.03 | 2.96 | 1.02 | 3.58 | 0.09 | n.d. | n.d. | 0.06 | 0.00 | 10.98 | 1.62 | 33.64 | 0.01 | 61.29 | 7.91 |
| CRL-50 | farmhouse | 0.37 | 0.01 | 2.38 | 0.58 | 3.35 | 0.29 | n.d. | n.d. | 0.08 | 0.02 | 7.79 | 0.42 | 32.43 | 4.32 | 39.49 | 2.01 |
| CRL-56 | farmhouse | 0.26 | n.d. | 1.45 | 0.46 | 2.40 | 0.08 | n.d. | n.d. | 0.07 | 0.01 | 7.46 | 0.12 | 47.73 | 4.83 | 31.48 | 0.64 |
| CRL-1 | lambic | 0.41 | 0.02 | 4.55 | 0.96 | 3.72 | 0.26 | 0.07 | 0.03 | 0.07 | 0.01 | 12.79 | 1.57 | 51.42 | 1.42 | 33.91 | 1.87 |
| CRL-4 | lambic | 0.60 | 0.03 | 3.31 | 0.69 | 2.05 | 0.15 | n.d. | n.d. | 0.05 | n.d. | 12.29 | 2.43 | 20.62 | 2.49 | 41.56 | 3.97 |
| CRL-5 | lambic | 1.16 | 0.73 | 5.06 | 1.80 | 2.87 | 0.22 | 0.09 | 0.01 | 0.07 | 0.01 | 13.66 | 3.42 | 47.37 | 7.79 | 40.15 | 4.83 |
| CRL-10 | lambic | 1.10 | 0.38 | 5.49 | 1.62 | 3.49 | 0.53 | n.d. | n.d. | 0.07 | 0.00 | 12.26 | 2.65 | 47.59 | 7.86 | 31.26 | 5.90 |
| CRL-15 | lambic | 0.36 | 0.03 | 2.46 | 0.87 | 2.77 | 0.26 | n.d. | n.d. | 0.07 | 0.01 | 11.72 | 1.91 | 46.08 | 10.16 | 51.56 | 1.54 |
| CRL-25 | lambic | 2.53 | 0.48 | 12.81 | 3.14 | 26.23 | 2.50 | 0.13 | n.d. | 0.31 | 0.08 | 16.73 | 1.70 | 76.11 | 13.15 | 220.83 | 13.80 |
| CRL-26 | lambic | 2.08 | 0.52 | 6.78 | 1.65 | 3.27 | 0.24 | n.d. | n.d. | 0.11 | 0.01 | 15.76 | 2.32 | 33.13 | 2.81 | 77.52 | 17.36 |
| CRL-27 | lambic | 0.55 | 0.02 | 11.13 | 1.69 | 4.04 | 0.47 | n.d. | n.d. | 0.10 | 0.01 | 25.25 | 4.13 | 46.74 | 17.49 | 80.62 | 15.91 |
| CRL-28 | lambic | 0.74 | 0.28 | 6.32 | 3.89 | 3.35 | 0.52 | 0.14 | 0.11 | 0.10 | 0.02 | 18.43 | 6.29 | 61.03 | 16.83 | 105.41 | 30.30 |
| CRL-29 | lambic | 0.98 | 0.17 | 10.77 | 1.32 | 8.40 | 2.49 | 0.21 | 0.02 | 0.13 | 0.03 | 19.64 | 1.61 | 85.96 | 11.07 | 151.07 | 20.14 |
| CRL-36 | lambic | 1.98 | 0.52 | 8.14 | 1.41 | 3.41 | 0.21 | 0.10 | 0.04 | 0.14 | 0.04 | 19.48 | 2.40 | 60.90 | 9.18 | 98.13 | 9.09 |
| CRL-55 | lambic | 0.28 | 0.01 | 6.54 | 0.33 | 3.03 | 0.10 | n.d. | n.d. | 0.07 | 0.01 | 16.17 | 1.97 | 39.50 | 2.22 | 57.78 | 1.48 |
| CRL-59 | lambic | n.d. | n.d. | 1.29 | 0.32 | 2.07 | 0.40 | n.d. | n.d. | n.d. | n.d. | 8.13 | 1.71 | 16.23 | 8.92 | 29.88 | 2.10 |
| CRL-91 | lambic | n.d. | n.d. | 1.12 | 0.11 | 1.71 | 0.11 | n.d. | n.d. | 0.07 | 0.01 | 5.00 | 0.87 | 18.63 | 1.72 | 24.74 | 6.29 |
| CRL-64 | naardensis | n.d. | n.d. | 0.65 | 0.16 | 4.16 | 1.04 | n.d. | n.d. | n.d. | n.d. | n.d. | n.d. | 21.59 | 8.80 | 3.41 | 0.42 |
| CRL-65 | naardensis | 0.58 | 0.37 | 1.21 | 0.14 | 1.73 | 0.14 | n.d. | n.d. | n.d. | n.d. | 4.66 | 1.03 | n.d. | n.d. | 5.18 | 0.57 |
| CRL-66 | naardensis | n.d. | n.d. | 3.00 | 0.17 | 1.84 | 0.04 | n.d. | n.d. | n.d. | n.d. | 6.51 | 0.87 | 22.15 | 1.25 | 7.21 | 0.68 |
| CRL-2 | wild | 0.64 | 0.25 | 2.82 | 0.21 | 3.20 | 0.25 | n.d. | n.d. | n.d. | n.d. | 9.02 | 0.07 | n.d. | n.d. | 15.20 | 3.25 |
| CRL-3 | wild | 1.33 | 0.14 | 3.25 | 0.11 | 3.14 | 0.12 | n.d. | n.d. | n.d. | n.d. | 11.20 | 1.26 | n.d. | n.d. | 17.49 | 6.95 |
| CRL-11 | wild | 0.35 | 0.07 | 1.97 | 0.64 | 2.25 | 0.20 | n.d. | n.d. | 0.06 | 0.01 | 8.99 | 2.34 | 21.97 | 3.42 | 13.11 | 5.31 |
| CRL-12 | wild | 0.64 | 0.09 | 1.54 | 0.12 | 3.19 | 0.24 | n.d. | n.d. | n.d. | n.d. | 8.37 | 0.47 | n.d. | n.d. | 10.77 | 2.63 |
| CRL-13 | wild | 0.28 | 0.02 | 1.78 | 0.53 | 2.22 | 0.27 | n.d. | n.d. | 0.05 | 0.00 | 9.04 | 2.66 | 20.28 | 3.06 | 17.84 | 2.47 |
| CRL-14 | wild | 1.01 | 0.89 | 2.03 | 0.35 | 3.68 | 0.50 | n.d. | n.d. | n.d. | n.d. | 9.96 | 1.96 | n.d. | n.d. | 37.36 | 8.03 |
| CRL-51 | wild | 0.27 | 0.01 | 1.63 | 0.25 | 2.06 | 0.05 | n.d. | n.d. | 0.06 | 0.00 | 8.78 | 1.08 | 15.20 | 2.93 | 25.88 | 3.70 |
| CRL-72 | wild | 0.34 | 0.04 | 0.76 | 0.11 | 1.23 | 0.02 | n.d. | n.d. | 0.05 | 0.01 | 5.04 | n.d. | 5.89 | n.d. | 6.76 | 2.44 |
| CRL-73 | wild | 0.27 | n.d. | 0.75 | 0.28 | 1.13 | 0.06 | n.d. | n.d. | 0.06 | 0.01 | 4.28 | n.d. | n.d. | n.d. | 7.02 | 3.16 |
| CRL-77 | wild | 0.96 | 0.30 | 2.16 | 0.21 | 2.09 | 0.10 | n.d. | n.d. | 0.05 | 0.00 | 7.16 | 1.41 | 5.49 | n.d. | 11.59 | 1.89 |
| CRL-80 | wild | 1.29 | 0.34 | 1.72 | 0.31 | 2.06 | 0.19 | n.d. | n.d. | 0.05 | 0.00 | 6.97 | 1.65 | n.d. | n.d. | 6.69 | 2.37 |
| CRL-8 | wine3n | 0.45 | 0.03 | 2.72 | 0.13 | 2.87 | 0.06 | n.d. | n.d. | 0.07 | 0.01 | 16.29 | 0.93 | 65.09 | 4.72 | 24.40 | 4.14 |
| CRL-22 | wine3n | 2.85 | 0.12 | 4.11 | 0.55 | 3.15 | 0.10 | n.d. | n.d. | 0.05 | 0.00 | 15.14 | 2.23 | 24.15 | 4.19 | 19.40 | 4.47 |
| CRL-23 | wine3n | 0.92 | 0.11 | 4.04 | 1.33 | 3.21 | 0.47 | n.d. | n.d. | 0.08 | 0.00 | 18.99 | 6.24 | 58.77 | 7.83 | 41.82 | 15.73 |
| CRL-24 | wine3n | 1.13 | 0.51 | 5.51 | 0.01 | 3.47 | 0.09 | n.d. | n.d. | 0.12 | 0.02 | 27.48 | 2.42 | 85.61 | 14.31 | 63.37 | 11.55 |
| CRL-75 | wine3n | n.d. | n.d. | 1.92 | 0.28 | 2.27 | 0.18 | n.d. | n.d. | 0.09 | 0.01 | 7.17 | 1.22 | 44.66 | 7.56 | 18.79 | 5.20 |
| CRL-76 | wine3n | 0.27 | n.d. | 1.23 | 0.24 | 1.97 | 0.14 | n.d. | n.d. | 0.08 | 0.01 | 8.20 | 1.10 | 43.94 | 3.42 | 6.35 | 2.35 |
| CRL-78 | wine3n | 0.27 | 0.01 | 1.72 | 0.35 | 1.95 | 0.14 | n.d. | n.d. | 0.06 | n.d. | 7.77 | 1.04 | n.d. | n.d. | 10.96 | 4.64 |
| CRL-82 | wine3n | 0.46 | 0.08 | 2.93 | 0.11 | 2.19 | 0.02 | n.d. | n.d. | 0.11 | 0.01 | 10.26 | 0.55 | 40.99 | 4.17 | 12.01 | 0.27 |
| CRL-84 | wine3n | 0.28 | 0.02 | 1.51 | 0.48 | 2.32 | 0.32 | n.d. | n.d. | 0.09 | 0.02 | 8.18 | 2.13 | 49.57 | 10.84 | 8.04 | 3.66 |
| CRL-85 | wine3n | 0.29 | 0.01 | 1.95 | 0.20 | 1.87 | 0.03 | n.d. | n.d. | 0.10 | 0.01 | 14.68 | 0.98 | 31.26 | 10.57 | 20.95 | 4.35 |
| CRL-86 | wine3n | 0.37 | 0.01 | 2.69 | 0.17 | 2.09 | 0.14 | n.d. | n.d. | 0.11 | 0.02 | 10.95 | 0.37 | 62.63 | 12.35 | 15.72 | 4.79 |
| CRL-87 | wine3n | 0.36 | 0.04 | 2.20 | 0.32 | 2.72 | 0.20 | n.d. | n.d. | 0.12 | 0.01 | 11.13 | 1.04 | 57.36 | 6.49 | 21.59 | 5.72 |
| CRL-88 | wine3n | 0.31 | 0.05 | 1.41 | 0.40 | 1.32 | 0.13 | n.d. | n.d. | 0.07 | 0.01 | 10.66 | 2.01 | 16.58 | 3.77 | 7.79 | 3.75 |
| CRL-89 | wine3n | 0.91 | 0.40 | 2.12 | 0.18 | 2.36 | 0.10 | 0.07 | n.d. | 0.12 | 0.02 | 7.02 | 1.77 | 24.29 | 9.07 | 29.40 | 5.55 |
| CRL-40 | wine2n | 0.31 | 0.03 | 2.97 | 0.33 | 2.87 | 0.05 | n.d. | n.d. | 0.07 | 0.02 | 9.74 | 0.47 | 35.80 | 0.90 | 27.01 | 1.96 |
| CRL-62 | wine2n | 0.30 | 0.02 | 4.03 | 1.03 | 3.20 | 0.40 | n.d. | n.d. | 0.10 | 0.02 | 12.53 | 0.91 | 30.03 | 5.03 | 64.81 | 5.69 |
| CRL-79 | wine2n | 0.32 | 0.04 | 2.11 | 0.45 | 2.06 | 0.10 | 0.11 | 0.06 | 0.09 | 0.02 | 8.04 | 1.09 | 27.80 | 10.74 | 66.35 | 10.21 |
| CRL-81 | wine2n | 2.10 | 1.00 | 4.61 | 1.24 | 2.45 | 0.11 | 0.17 | 0.07 | 0.11 | 0.01 | 11.85 | 0.77 | 34.24 | 0.58 | 31.78 | 0.69 |
| CRL-83 | wine2n | 0.29 | 0.01 | 2.51 | 0.43 | 2.22 | 0.10 | n.d. | n.d. | 0.14 | 0.00 | 10.50 | 1.54 | 74.51 | 2.68 | 19.79 | 1.17 |
| CRL-96 | wine2n | 0.49 | 0.23 | 2.53 | 0.86 | 2.59 | 0.19 | 0.08 | 0.01 | 0.13 | 0.01 | 8.00 | 1.39 | 22.41 | 8.95 | 60.97 | 9.95 |

Table S3. Results of in vitro phenotypical assays of *Brettanomyces* collection. STDV (n=3).

|  |  | **Acetic acid** | | **Ethanol** | | **Growth in cellobiose** | | **Beta-glucosidase assay** | | **Growth in maltose** | | **Ferulic acid consumption** | |
| --- | --- | --- | --- | --- | --- | --- | --- | --- | --- | --- | --- | --- | --- |
| **CRL num** | **Cluster** | **gr/L** | **STDV** | **%v/v** | **STDV** | **OD600** | **STDV** | **U/L** | **STDV** | **B value** | **STDV** | **mg** | **STDV** |
| CRL-6 | - | 9,49 | 3,09 | 2,10 | 0,59 | 0,98 | 0,12 | -0,05 | 0,03 | 77,00 | 7,85 | 0,10 | 0,00 |
| CRL-52 | - | 13,22 | 1,16 | 1,57 | 0,40 | 1,76 | 0,05 | 2,64 | 0,49 | 59,00 | 1,00 | 0,10 | 0,00 |
| CRL-60 | - | 13,75 | 7,16 | 1,96 | 0,39 | 0,94 | 0,10 | -0,06 | 0,01 | 130,67 | 5,51 | 0,10 | 0,00 |
| CRL-63 | - | 15,31 | 6,39 | 1,05 | 0,40 | 1,94 | 0,06 | 0,47 | 0,17 | 72,67 | 25,58 | 0,10 | 0,00 |
| CRL-7 | anomalus | 11,33 | 2,18 | 1,76 | 0,63 | 1,89 | 0,01 | 1,05 | 0,13 | 84,00 | 26,89 | 0,10 | 0,00 |
| CRL-18 | anomalus | 9,27 | 0,51 | 1,62 | 0,17 | 1,93 | 0,01 | 0,15 | 0,04 | 14,00 | 19,60 | 0,10 | 0,00 |
| CRL-30 | anomalus | 7,81 | 1,60 | 1,58 | 0,09 | 0,84 | 0,12 | -0,04 | 0,03 | 110,67 | 22,43 | 0,10 | 0,00 |
| CRL-39 | anomalus | 8,96 | 0,76 | 0,92 | 0,33 | 1,92 | 0,00 | 0,26 | 0,01 | 76,00 | 52,79 | 0,10 | 0,00 |
| CRL-41 | anomalus | 10,95 | 0,54 | 1,34 | 0,26 | 1,86 | 0,02 | 0,20 | 0,03 | 0,67 | 22,07 | 0,10 | 0,00 |
| CRL-49 | anomalus | 10,31 | 2,05 | 1,73 | 0,52 | 1,94 | 0,01 | 0,31 | 0,06 | 116,00 | 13,27 | 0,10 | 0,00 |
| CRL-57 | anomalus | 9,38 | 3,51 | 1,50 | 0,15 | 1,86 | 0,02 | 0,47 | 0,02 | 144,67 | 1,25 | 0,10 | 0,00 |
| CRL-58 | anomalus | 19,56 | 7,95 | 1,53 | 0,02 | 1,88 | 0,02 | 0,14 | 0,02 | 131,83 | 1,50 | 0,10 | 0,00 |
| CRL-61 | anomalus | 11,81 | 0,06 | 1,12 | 0,11 | 1,87 | 0,01 | -0,06 | 0,05 | 122,83 | 4,50 | 0,10 | 0,00 |
| CRL-67 | anomalus | 9,78 | 1,75 | 1,49 | 0,11 | 1,92 | 0,05 | 0,24 | 0,06 | 68,33 | 13,00 | 0,10 | 0,00 |
| CRL-68 | anomalus | 11,57 | 0,15 | 1,36 | 0,02 | 1,85 | 0,07 | 0,61 | 0,26 | 85,00 | 15,06 | 0,10 | 0,00 |
| CRL-69 | anomalus | 11,85 | 0,55 | 1,26 | 0,17 | 2,18 | 0,06 | 0,23 | 0,17 | 135,33 | 17,38 | 0,08 | 0,03 |
| CRL-74 | anomalus | 14,51 | 2,13 | 1,82 | 0,09 | 1,92 | 0,01 | 0,16 | 0,05 | 134,67 | 18,26 | 0,10 | 0,00 |
| CRL-90 | anomalus | 12,46 | 0,83 | 1,71 | 0,34 | 1,89 | 0,06 | 0,71 | 0,05 | -22,67 | 0,00 | 0,03 | 0,00 |
| CRL-42 | custersianus | 7,05 | 2,12 | 0,57 | 0,15 | 1,42 | 0,43 | 0,72 | 0,55 | 44,67 | 11,15 | 0,00 | 0,01 |
| CRL-53 | custersianus | 6,26 | 1,59 | 0,96 | 0,46 | 1,30 | 0,32 | 0,30 | 0,28 | 46,00 | 22,16 | 0,00 | 0,03 |
| CRL-54 | custersianus | 5,66 | 1,07 | 0,76 | 0,29 | 1,77 | 0,47 | 0,69 | 0,17 | 31,33 | 2,94 | 0,00 | 0,01 |
| CRL-9 | farmhouse | 9,52 | 0,72 | 1,51 | 0,04 | 1,08 | 0,10 | 0,01 | 0,06 | 59,33 | 15,51 | 0,10 | 0,00 |
| CRL-16 | farmhouse | 16,39 | 5,84 | 1,44 | 0,59 | 1,19 | 0,04 | -0,06 | 0,17 | 62,33 | 21,46 | 0,10 | 0,00 |
| CRL-17 | farmhouse | 17,89 | 1,98 | 1,42 | 0,37 | 1,89 | 0,05 | 0,10 | 0,02 | 7,50 | 3,54 | 0,10 | 0,00 |
| CRL-19 | farmhouse | 13,03 | 4,20 | 1,98 | 0,73 | 2,04 | 0,30 | 0,12 | 0,17 | 12,67 | 23,03 | 0,10 | 0,00 |
| CRL-20 | farmhouse | 11,24 | 1,08 | 1,71 | 0,53 | 1,06 | 0,13 | 0,04 | 0,30 | -5,00 | 16,37 | 0,10 | 0,00 |
| CRL-21 | farmhouse | 11,62 | 1,89 | 1,58 | 0,44 | 1,23 | 0,20 | -0,13 | 0,09 | 33,00 | 25,71 | 0,10 | 0,00 |
| CRL-31 | farmhouse | 12,62 | 0,19 | 1,80 | 0,21 | 1,20 | 0,16 | -0,07 | 0,09 | 87,67 | 27,68 | 0,10 | 0,00 |
| CRL-33 | farmhouse | 12,50 | 3,35 | 1,93 | 0,66 | 1,14 | 0,04 | -0,24 | 0,08 | 68,00 | 32,92 | 0,10 | 0,00 |
| CRL-34 | farmhouse | 11,60 | 1,92 | 1,66 | 0,12 | 1,06 | 0,03 | -0,14 | 0,11 | 6,67 | 17,90 | 0,10 | 0,00 |
| CRL-35 | farmhouse | 13,44 | 2,27 | 2,38 | 0,56 | 1,03 | 0,07 | -0,03 | 0,04 | 29,00 | 27,00 | 0,10 | 0,00 |
| CRL-38 | farmhouse | 13,22 | 0,50 | 1,31 | 0,28 | 1,23 | 0,04 | -0,13 | 0,23 | 40,33 | 7,57 | 0,10 | 0,00 |
| CRL-47 | farmhouse | 8,96 | 1,07 | 1,72 | 0,38 | 0,78 | 0,23 | -0,06 | 0,05 | 83,33 | 5,51 | 0,10 | 0,00 |
| CRL-48 | farmhouse | 16,92 | 2,55 | 1,43 | 0,90 | 1,68 | 0,25 | -0,02 | 0,19 | 83,00 | 20,22 | 0,10 | 0,01 |
| CRL-50 | farmhouse | 12,63 | 1,35 | 1,22 | 0,18 | 1,05 | 0,08 | -0,42 | 0,65 | 82,67 | 21,13 | 0,10 | 0,00 |
| CRL-56 | farmhouse | 17,42 | 4,57 | 1,36 | 0,30 | 1,14 | 0,17 | -0,10 | 0,11 | 74,00 | 16,52 | 0,10 | 0,00 |
| CRL-1 | lambic | 9,19 | 2,54 | 1,57 | 0,16 | 0,94 | 0,08 | -0,22 | 0,06 | 106,33 | 26,47 | 0,09 | 0,02 |
| CRL-4 | lambic | 11,21 | 3,15 | 1,45 | 0,50 | 0,88 | 0,06 | -0,13 | 0,13 | 98,33 | 13,88 | 0,10 | 0,00 |
| CRL-5 | lambic | 10,27 | 0,26 | 1,56 | 0,20 | 1,07 | 0,10 | -0,31 | 0,46 | 54,33 | 25,51 | 0,10 | 0,00 |
| CRL-10 | lambic | 17,32 | 4,60 | 2,59 | 0,83 | 0,89 | 0,08 | -0,17 | 0,13 | 65,67 | 27,35 | 0,10 | 0,00 |
| CRL-15 | lambic | 14,02 | 1,51 | 1,21 | 0,43 | 0,98 | 0,16 | -0,11 | 0,02 | 64,67 | 22,12 | 0,10 | 0,01 |
| CRL-25 | lambic | 9,41 | 1,60 | 2,22 | 0,48 | 0,49 | 0,11 | -0,08 | 0,03 | 83,67 | 28,38 | 0,10 | 0,00 |
| CRL-26 | lambic | 8,85 | 1,20 | 1,72 | 0,89 | 0,58 | 0,25 | -0,14 | 0,01 | 43,50 | 45,96 | 0,10 | 0,00 |
| CRL-27 | lambic | 10,21 | 3,84 | 1,23 | 0,11 | 0,94 | 0,13 | -0,34 | 0,05 | 43,33 | 11,85 | 0,10 | 0,00 |
| CRL-28 | lambic | 10,17 | 0,53 | 2,12 | 0,59 | 0,95 | 0,04 | -0,18 | 0,12 | 91,33 | 27,43 | 0,10 | 0,00 |
| CRL-29 | lambic | 10,57 | 1,17 | 2,04 | 0,14 | 0,52 | 0,05 | -0,12 | 0,01 | 50,00 | 32,19 | 0,08 | 0,03 |
| CRL-36 | lambic | 9,13 | 0,06 | 1,11 | 0,06 | 0,79 | 0,06 | -0,08 | 0,01 | 46,67 | 41,63 | 0,10 | 0,00 |
| CRL-55 | lambic | 13,72 | 1,07 | 1,42 | 0,44 | 0,89 | 0,05 | -0,05 | 0,01 | 53,33 | 34,93 | 0,10 | 0,01 |
| CRL-59 | lambic | 16,33 | 4,76 | 1,92 | 0,71 | 1,49 | 0,08 | -0,15 | 0,10 | 78,33 | 2,52 | 0,08 | 0,02 |
| CRL-91 | lambic | 7,89 | 2,16 | 1,41 | 0,35 | 1,32 | 0,34 | 0,04 | 0,02 | 72,67 | 49,89 | 0,10 | 0,00 |
| CRL-64 | naardensis | 10,16 | 6,94 | 0,85 | 0,12 | 1,88 | 0,07 | 0,09 | 0,17 | 67,00 | 5,19 | 0,01 | 0,01 |
| CRL-65 | naardensis | 8,50 | 0,34 | 0,76 | 0,18 | 1,89 | 0,08 | -0,24 | 0,01 | -0,67 | 4,32 | 0,09 | 0,02 |
| CRL-66 | naardensis | 7,48 | 1,46 | 0,96 | 0,18 | 1,76 | 0,21 | 0,18 | 0,09 | 44,00 | 22,31 | 0,00 | 0,01 |
| CRL-2 | wild | 13,79 | 0,91 | 1,63 | 0,28 | 1,70 | 0,06 | 0,03 | 0,05 | -22,67 | 0,00 | 0,10 | 0,00 |
| CRL-3 | wild | 12,29 | 0,69 | 1,43 | 0,32 | 1,50 | 0,07 | 0,01 | 0,04 | -22,67 | 0,00 | 0,10 | 0,00 |
| CRL-11 | wild | 13,00 | 1,30 | 1,30 | 0,05 | 1,70 | 0,09 | 0,40 | 0,20 | -15,67 | 4,97 | 0,10 | 0,00 |
| CRL-12 | wild | 17,18 | 9,53 | 1,18 | 0,14 | 1,44 | 0,01 | -0,01 | 0,01 | -15,67 | 9,90 | 0,10 | 0,00 |
| CRL-13 | wild | 14,07 | 0,12 | 1,98 | 0,02 | 1,52 | 0,11 | 0,29 | 0,35 | -18,67 | 4,97 | 0,10 | 0,00 |
| CRL-14 | wild | 16,75 | 4,64 | 0,85 | 0,17 | 1,79 | 0,05 | -0,28 | 0,07 | 9,00 | 30,05 | 0,10 | 0,00 |
| CRL-51 | wild | 16,84 | 3,30 | 1,40 | 0,34 | 1,92 | 0,01 | 0,04 | 0,03 | 51,00 | 4,36 | 0,10 | 0,00 |
| CRL-72 | wild | 9,78 | 3,60 | 1,30 | 0,67 | 1,84 | 0,21 | 0,01 | 0,02 | 17,33 | 23,16 | 0,09 | 0,03 |
| CRL-73 | wild | 12,25 | 1,19 | 1,66 | 0,48 | 1,91 | 0,25 | -0,17 | 0,21 | 21,00 | 36,76 | 0,10 | 0,00 |
| CRL-77 | wild | 8,67 | 0,54 | 1,32 | 0,36 | 1,77 | 0,01 | 0,64 | 0,21 | 16,33 | 6,03 | 0,10 | 0,00 |
| CRL-80 | wild | 9,83 | 2,23 | 1,12 | 0,19 | 1,61 | 0,03 | 0,16 | 0,02 | -6,67 | 5,51 | 0,10 | 0,00 |
| CRL-8 | wine3n | 11,22 | 1,47 | 1,61 | 0,50 | 1,86 | 0,01 | 0,13 | 0,08 | 48,33 | 6,48 | 0,10 | 0,00 |
| CRL-22 | wine3n | 7,82 | 1,51 | 1,45 | 0,52 | 1,10 | 0,41 | 0,13 | 0,19 | 6,50 | 9,19 | 0,03 | 0,03 |
| CRL-23 | wine3n | 16,08 | 5,33 | 1,38 | 0,24 | 1,69 | 0,02 | 1,42 | 0,65 | 40,33 | 22,23 | 0,10 | 0,00 |
| CRL-24 | wine3n | 11,88 | 1,14 | 1,50 | 0,39 | 0,45 | 0,06 | 1,03 | 0,22 | 33,33 | 14,84 | 0,10 | 0,00 |
| CRL-75 | wine3n | 14,81 | 6,83 | 1,53 | 0,04 | 1,85 | 0,09 | 0,02 | 0,06 | -2,00 | 0,00 | 0,10 | 0,00 |
| CRL-76 | wine3n | 6,95 | 2,28 | 1,17 | 0,17 | 1,97 | 0,13 | 0,34 | 0,10 | 94,00 | 10,39 | 0,07 | 0,06 |
| CRL-78 | wine3n | 7,78 | 1,98 | 1,18 | 0,06 | 1,71 | 0,02 | -0,03 | 0,03 | 100,33 | 15,37 | 0,10 | 0,00 |
| CRL-82 | wine3n | 5,68 | 1,83 | 0,98 | 0,33 | 1,82 | 0,01 | 0,56 | 0,03 | -7,00 | 8,66 | 0,10 | 0,00 |
| CRL-84 | wine3n | 7,57 | 1,16 | 0,90 | 0,28 | 1,96 | 0,03 | 0,19 | 0,08 | 10,00 | 32,23 | 0,10 | 0,00 |
| CRL-85 | wine3n | 8,08 | 2,23 | 1,50 | 0,13 | 1,50 | 0,06 | 0,13 | 0,04 | 59,33 | 64,69 | 0,08 | 0,02 |
| CRL-86 | wine3n | 7,02 | 1,47 | 1,24 | 0,13 | 1,63 | 0,04 | 0,34 | 0,09 | 99,67 | 86,32 | 0,09 | 0,02 |
| CRL-87 | wine3n | 6,99 | 1,90 | 1,00 | 0,38 | 1,47 | 0,03 | 0,11 | 0,05 | 80,00 | 46,13 | 0,08 | 0,01 |
| CRL-88 | wine3n | 7,76 | 1,03 | 1,38 | 0,52 | 1,61 | 0,02 | 0,07 | 0,03 | 140,67 | 18,50 | 0,10 | 0,00 |
| CRL-89 | wine3n | 5,37 | 1,01 | 1,40 | 0,23 | 1,56 | 0,07 | 0,14 | 0,03 | -12,00 | 0,00 | 0,10 | 0,00 |
| CRL-40 | wine2n | 14,65 | 3,07 | 2,10 | 1,35 | 2,14 | 0,23 | 0,26 | 0,58 | -17,33 | 2,31 | 0,08 | 0,03 |
| CRL-62 | wine2n | 17,58 | 4,36 | 1,15 | 0,14 | 1,44 | 0,07 | 0,05 | 0,08 | 40,00 | 25,24 | 0,09 | 0,01 |
| CRL-79 | wine2n | 10,50 | 1,41 | 1,52 | 0,04 | 1,91 | 0,01 | 0,01 | 0,01 | 35,00 | 35,36 | 0,10 | 0,05 |
| CRL-81 | wine2n | 7,16 | 0,91 | 1,38 | 0,31 | 1,57 | 0,06 | 0,09 | 0,01 | -11,67 | 0,58 | 0,10 | 0,00 |
| CRL-83 | wine2n | 10,49 | 0,03 | 1,35 | 0,28 | 1,80 | 0,01 | 0,46 | 0,05 | -11,00 | 1,41 | 0,10 | 0,00 |
| CRL-96 | wine2n | 8,25 | 0,45 | 1,68 | 0,23 | 1,66 | 0,07 | 0,37 | 0,21 | -8,67 | 5,77 | 0,10 | 0,00 |

| **Coding sequence** | **Function – closest hit** | **Accession - closest hit** |
| --- | --- | --- |
| g4098 | TPA_exp: Fructosyl amine: oxygen oxidoreductase [Trichophyton benhamiae CBS 112371]; | DAA74868.1 |
| g4099 | Piso0_003683 [Millerozyma farinosa CBS 7064]; | CCE84142.1 |
| g4100 | major facilitator superfamily transporter [Brettanomyces bruxellensis AWRI1499]; | EIF45168.1 |
| g4101 | d-lactate dehydrogenase [Brettanomyces bruxellensis AWRI1499]; | EIF45169.1 |
| g4102 | zn 2cys6 transcription factor [Brettanomyces bruxellensis AWRI1499]; | EIF45174.1 |
| g4103 | multidrug-resistance transporter [Brettanomyces bruxellensis AWRI1499]; | EIF45173.1 |
| g4104 | 2cys6 transcription factor [Brettanomyces bruxellensis AWRI1499] | EIF45174.1 |
| g4105 | multidrug-resistance transporte [Brettanomyces bruxellensis AWRI1499]; | EIF45175.1 |
| g4106 | K7_Ami1p [Saccharomyces cerevisiae Kyokai no. 7]; | GAA26168.1 |
| g4107 | phenolic acid decarboxylase [Brettanomyces bruxellensis]; | APP94188.1 |
| g4108 | hypothetical protein AWRI1499_4964 [Brettanomyces bruxellensis AWRI1499]; | EIF45179.1 |
| g4109 | hexose transporter [Brettanomyces bruxellensis AWRI1499]; | EIF48172.1 |
| g4110 | unnamed protein product [Kluyveromyces marxianus]; | CAA29353.1 |
| g4111 | LAMI_0B05996g1_1 [Lachancea mirantina]; | SCU81385.1 |

Table S4. Predicted coding sequences missing in the same scaffold where DaPAD1 is located. The region was predicted with BLAST of CRL-49_0685 scaffold against the genome of CRL-90.


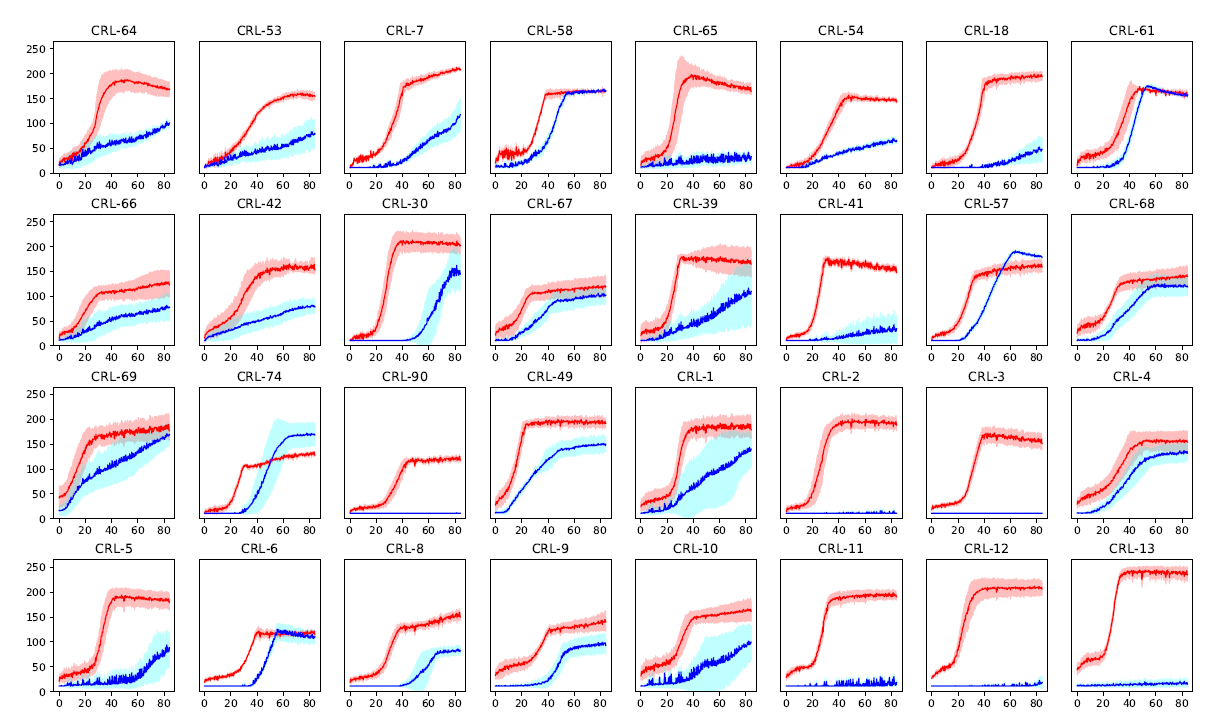


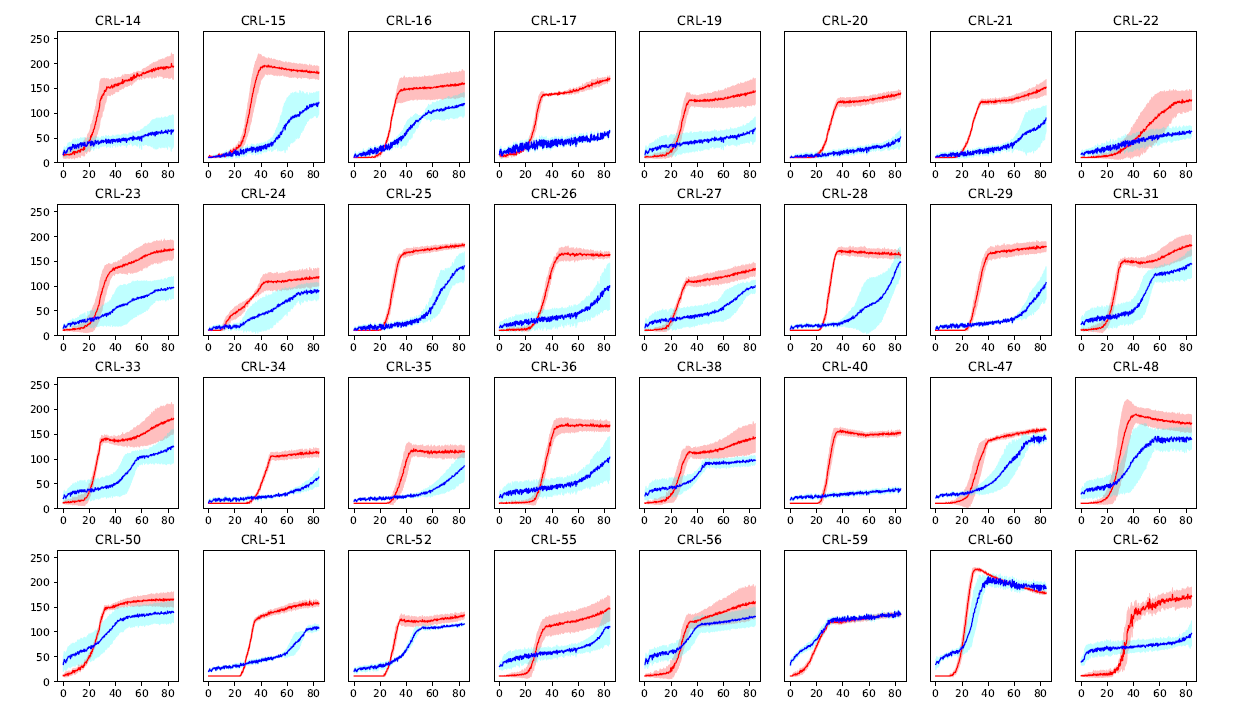


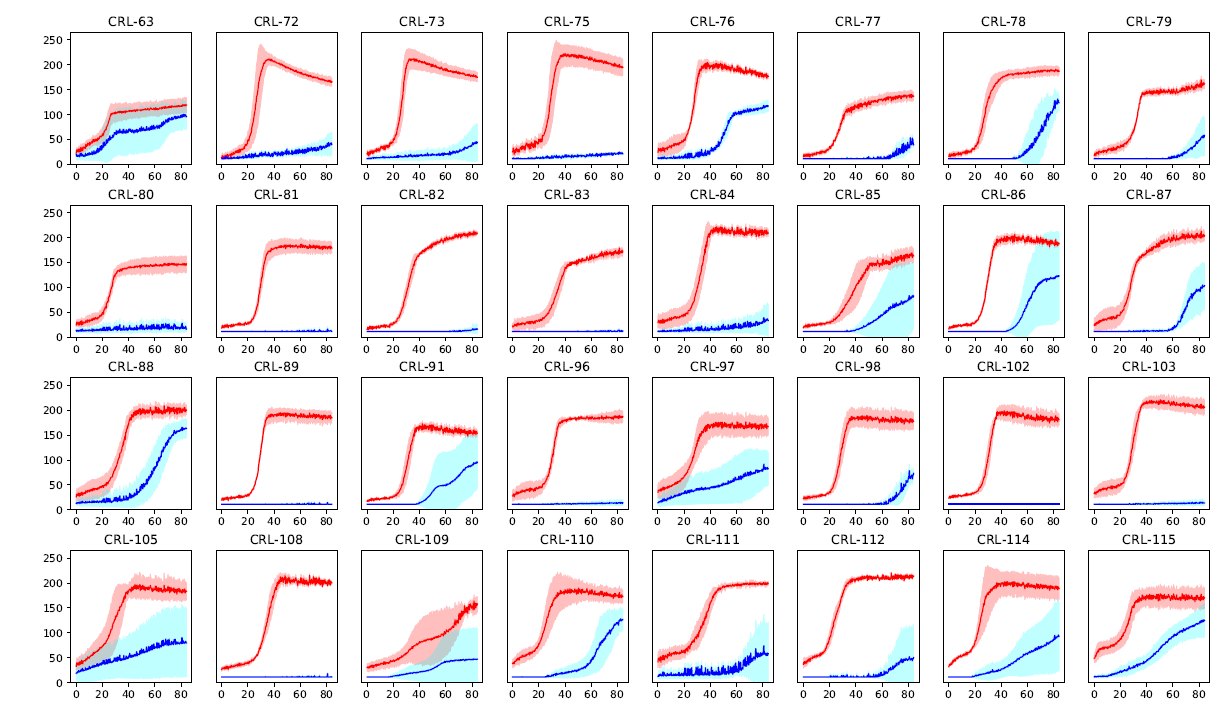


Figure S1. Growth of *Brettanomyces* strains in synthetic media with glucose (red) or maltose (blue). Standard deviation is indicated with curve shading. Y axis: Biolog value; X axis: time (hours)


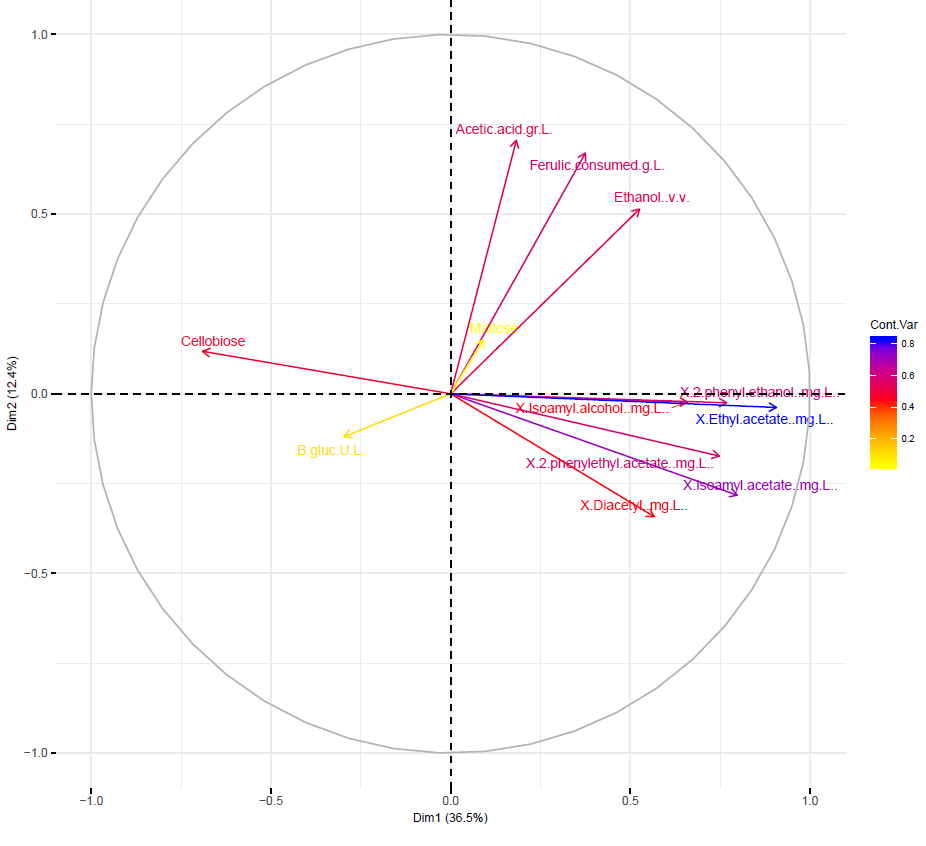


Figure S2. Loading of each phenotypical variable into dimensions 1 and 2 of the Principal Component Analysis.


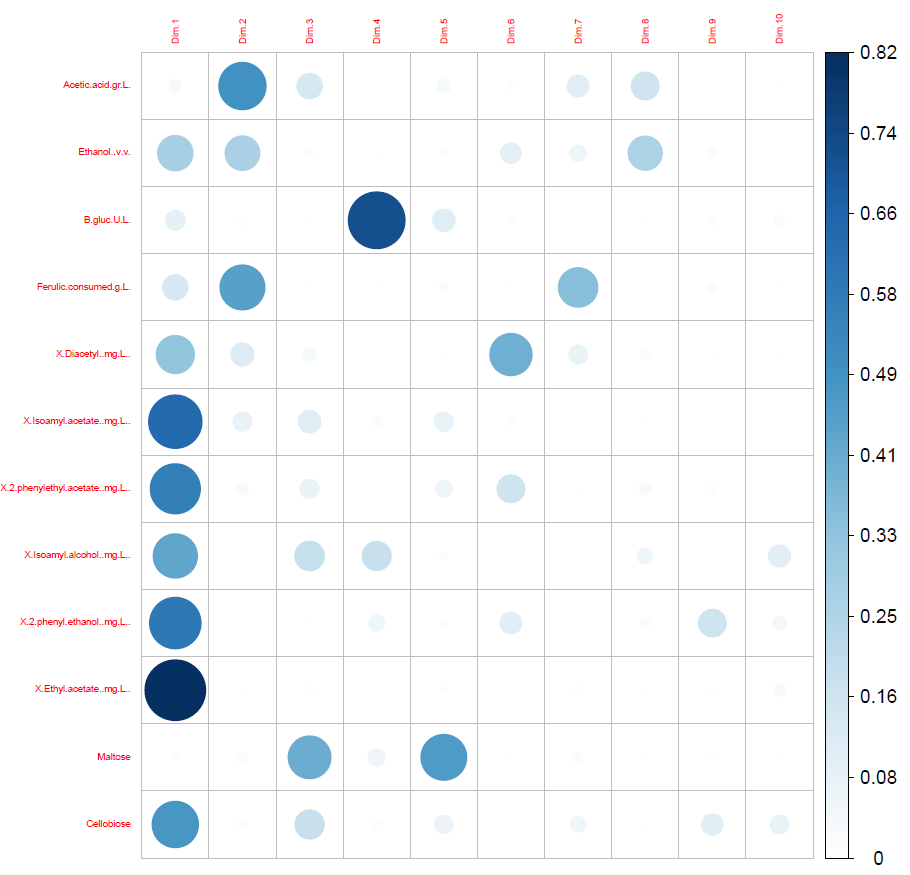


Figure S3. Loading of each phenotypical variable into 10 dimensions of the Principal Component Analysis.


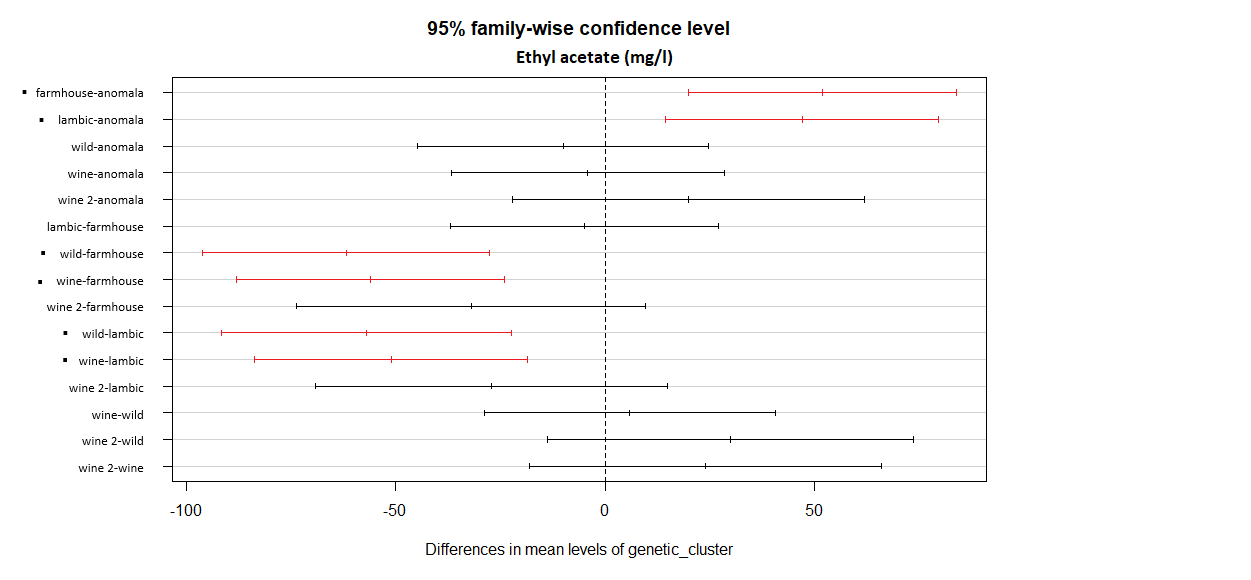


Statistical test 1. Comparison of ethyl acetate production of different genomic clusters. Tukey Post Hoc statistical test showing 95% family-wise confidence level. Comparisons resulting in significant differences are colored in red.


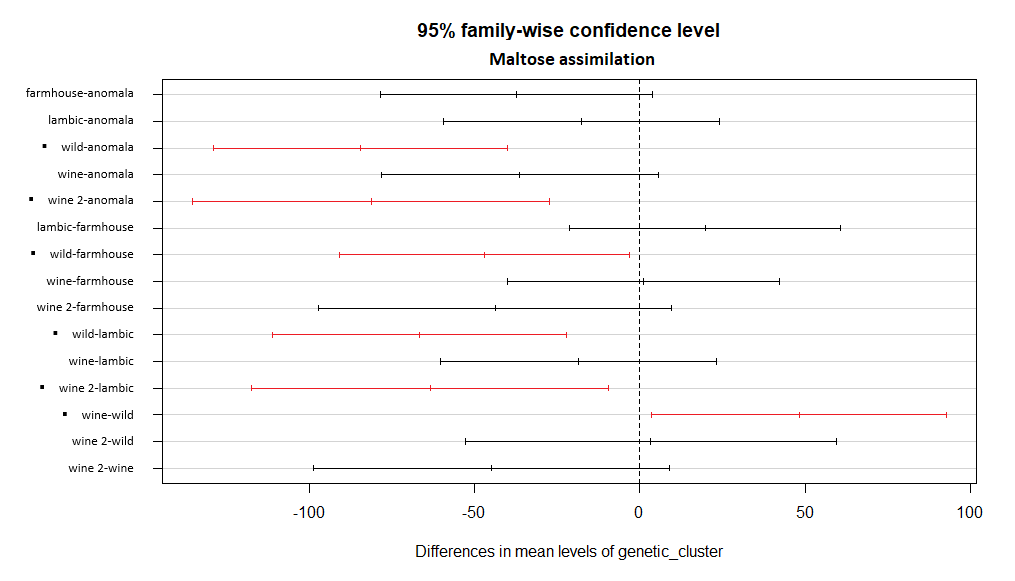


Statistical test 2. Comparison of maltose assimilation in the different genomic clusters. Tukey Post Hoc statistical test showing 95% family-wise confidence level. Comparisons resulting in significant differences are colored in red.


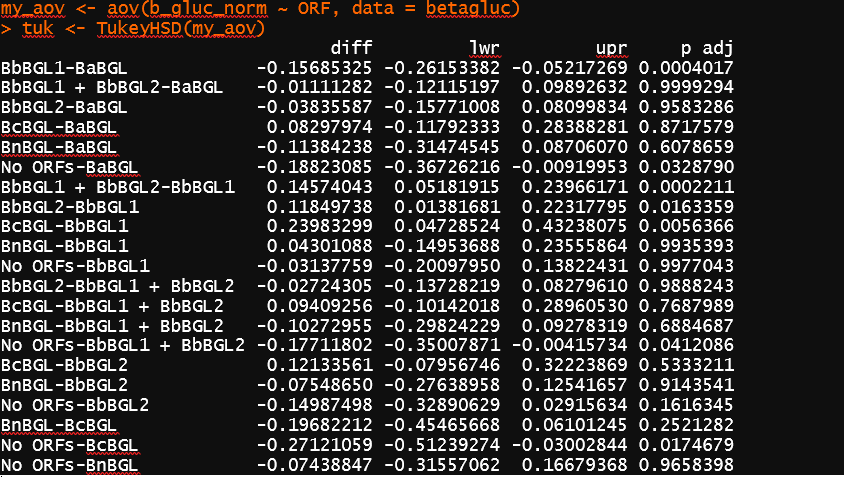


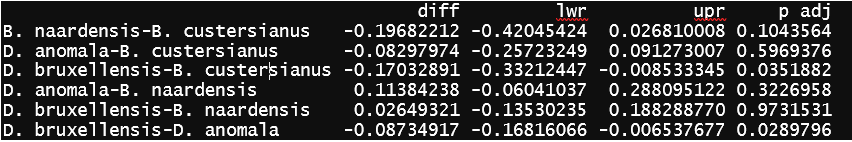


Statistical test 3. Comparison of the beta-glucosidase activity of *Brettanomyces* strains. At the top, comparison of strains according to its ORF coding for beta-glucosidase. At the bottom, comparison of strains based on species. Comparison using Tukey Posthoc test.
